# Spatial Analysis of Agricultural Waste and By‐Products to Tackle the Water–Energy Nexus in Rural Mozambique

**DOI:** 10.1002/gch2.202500339

**Published:** 2025-11-29

**Authors:** Giuseppe Mancuso, Valentina Morini, Gonzalo A. Martinez, Attilio Toscano, Francesca Valenti

**Affiliations:** ^1^ Department of Agricultural and Food Sciences Alma Mater Studiorum ‐ University of Bologna Bologna Italy; ^2^ Department of Civil Chemical Environmental and Materials Engineering Alma Mater Studiorum ‐ University of Bologna Bologna Italy

**Keywords:** agricultural water management, bioenergy, circular economy, GIS, sustainability, waste‐to‐resource, WEF nexus

## Abstract

Addressing energy and water management in rural Mozambique is essential for sustainable agricultural development. This study focuses on Nampula Province, where limited access to these resources deepens socioeconomic and environmental challenges. The research promotes sustainability by identifying, planning, and implementing innovative and socially validated solutions to enhance the water‐energy nexus for agricultural growth. In this study, an integrated approach combining geographic information system (GIS) tools and participatory methods is developed to assess and address local needs. The initial phase involved analyzing the rural context through field surveys, stakeholder interviews, community workshops, and site visits to collect and validate data, using tailored questionnaires and digital platforms. In the second phase, collected data are processed using GIS, building a geodatabase with layers such as land use, crop distribution, water demand, energy needs, and locations of processing facilities. QGIS software is used to map resource potential, deficits, and spatial disparities. These analyses provide key insights to guide sustainable interventions, helping identify critical areas and opportunities for optimizing resource use. This integrated and participatory approach can efficiently ensure the development of solutions that are contextually appropriate, technically robust, and socially validated, thereby laying the groundwork for effective and sustainable resource management strategies in Nampula.

## Graphical abstract

This study presents a geographic information system‐based, participatory methodology to identify high‐potential zones for agricultural waste valorization in rural Mozambique. By integrating spatial data with local knowledge, it reveals opportunities for bioenergy production and sustainable water use. The findings support circular development strategies, addressing the water–energy nexus while promoting inclusive rural resilience and alignment with global cooperation agendas.

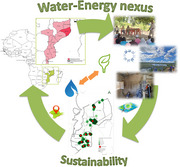

## 1 Introduction

Access to water and energy remains one of the most pressing challenges in many African countries, particularly in Mozambique, where large segments of the population still lack reliable, affordable, and sustainable services [1, 2].

The interdependence between water and energy, commonly referred to as the water‐energy nexus, is further exacerbated by climate vulnerability, institutional fragmentation, and pronounced socioeconomic inequalities. Tackling this nexus requires integrated, low‐cost, and context‐specific solutions that are both technically feasible and socially acceptable [3, 4].

In this context, the transition toward a circular economy offers a strategic opportunity to mitigate environmental degradation while simultaneously enhancing resource efficiency, promoting local empowerment, and fostering climate resilience. The valorization of agricultural residues and by‐products, such as their transformation into bioenergy, biofertilizers, or soil amendments, can play a key role in improving access to clean energy, reducing pressure on ecosystems, and strengthening rural livelihoods [5]. Recent studies have explored the potential of food waste‐derived biochar for environmental remediation, particularly in removing heavy metals from wastewater, highlighting additional opportunities for integrating agricultural by‐products into sustainable resource management [6]. Likewise, circular approaches to water management [7], such as wastewater reuse [8], rainwater harvesting [9, 10], and the integration of water‐saving practices in agriculture [11], proved to enhance water security, reduce environmental impacts, and support sustainable food systems. This study contributes to the literature by combining geographic information system (GIS)‐based spatial analysis with participatory methods to identify priority areas for agricultural waste valorization and integrated water management in Mozambique. The originality lies in the integration of technical (spatial analysis) and social (stakeholder engagement) dimensions to produce a replicable framework that informs both sustainable rural development and local policy‐making. These solutions are also connected to global sustainability frameworks, including the European Green Deal. To clarify its relevance in the African context, the European Green Deal promotes cooperation with African countries through funding opportunities (e.g., investment in renewable energy and sustainable infrastructure), policy alignment (particularly regarding climate adaptation and circular economy strategies), and partnership mechanisms. Such instruments can support African countries, including Mozambique, in implementing context‐sensitive, locally grounded solutions while benefiting from international collaboration and resources [11].

Furthermore, there is growing international interest in fostering more equitable, mutually beneficial partnerships with African countries. Initiatives such as the European Union–Africa Strategy and the recently launched Mattei Plan by the Italian government have placed Africa at the center of a renewed vision for cooperation. These programs emphasize shared growth, investment in sustainable infrastructure, and support for local development solutions, especially in strategic sectors such as energy, water, and agriculture. The Mattei Plan, in particular, highlights inclusive development through concrete, community‐oriented projects and the valorization of local resources, offering opportunities for policy integration with Mozambique's rural development strategies.

Mozambique, with its predominantly agrarian economy, generates large volumes of agricultural waste, particularly from the cultivation of maize, cassava, sugarcane, and rice [12]. However, these biomasses are often underutilized or poorly managed, contributing to greenhouse gas (GHG) emissions, soil degradation, and health risks in rural and peri‐urban areas. Moreover, understanding the nexus between agricultural practices and GHG emissions is critical for designing sustainable interventions. Using approaches such as the C‐vine Copula model, Pakrooh et al. [13] provide new insights into how AFOLU (Agriculture, Forestry, and Other Land Use) activities contribute to emissions, underscoring the importance of linking environmental and socioeconomic objectives [13]. At the same time, agricultural activities heavily rely on water resources, which are increasingly affected by unpredictable rainfall, droughts, and limited irrigation infrastructure. Inefficient water use and lack of integrated water management further constrain productivity and resilience in rural areas. In addition, novel methods such as interval meta‐goal programming have been applied to optimize agricultural water‐land use, supporting sustainable planning that integrates productivity, resource efficiency, and environmental goals [14]. This complements circular approaches by providing quantitative frameworks for decision‐making in resource‐constrained contexts.

To unlock the potential of agricultural waste and by‐products valorization, a spatially explicit understanding of the quantity and distribution of biomass flow is essential. Such an understanding supports not only the identification of key waste‐generation zones but also the assessment of territorial constraints, including infrastructure gaps, accessibility issues, and levels of social vulnerability. Equally important is the spatial characterization of water availability and usage patterns, as water scarcity or uneven distribution can significantly influence the sustainability of agricultural systems.

Territorial and spatial analyses, supported by GIS, have proven effective in identifying renewable energy potential and siting appropriate technologies, while also enabling the assessment of water availability, thus supporting integrated planning across various African contexts [15, 16, 17, 18].

Although GIS‐based methods have previously been applied in Mozambique to assess biomass energy potential [2], map irrigation systems [19], and analyze urban land‐use dynamics [20], a critical research gap exists: territorial analysis that simultaneously valorizes agricultural waste, supports decentralized energy production, enhances soil fertility, and integrates water resource management within a participatory framework. By addressing this gap, the current study advances the field by combining technical spatial methods with stakeholder engagement, aligning with emerging research on integrated resource management in sub‐Saharan Africa. This study addresses this gap by developing a GIS‐based methodology to quantify and map agricultural waste and by‐products in Nampula Province, integrating spatial datasets (agricultural production, land use, population density, infrastructure, water availability, and environmental constraints) with socioeconomic indicators and expert knowledge. Stakeholders consultations and participatory methods were employed to ensure that identified priority areas are socially relevant, technically feasible, and policy actionable. In parallel, qualitative fieldwork, such as semi‐structured interviews with local farmers, cooperatives, and agricultural processing industries, were used to provide critical insights into existing waste management practices, production constraints, and attitudes toward the adoption of innovative technologies. This ensures that the spatial analysis remains grounded in local realities and responsive to community needs.

By explicitly linking local participatory insights with spatially explicit quantitative models, the study contributes novel evidence to the literature on rural resource management, demonstrating how GIS‐based methods can inform actionable, context‐sensitive interventions. The results provide actionable insights for policymakers, NGOs, and development actors, facilitating low‐cost, locally appropriate interventions for agricultural waste valorization and sustainable water management. Moreover, the GIS‐based approach presented in this study offers a scalable and replicable framework for other African countries facing similar challenges. In doing so, it advances existing research by contextualizing Mozambique's experience within broader debates on the circular economy, climate adaptation, and integrated rural development in sub‐Saharan Africa. By repositioning agricultural waste as a resource rather than a burden, and by aligning spatial planning with community action, this research supports Mozambique's transition toward a more sustainable, circular, and inclusive development trajectory.

## 2 Materials and Methods

### 2.1 Study Area

**FIGURE 1:**
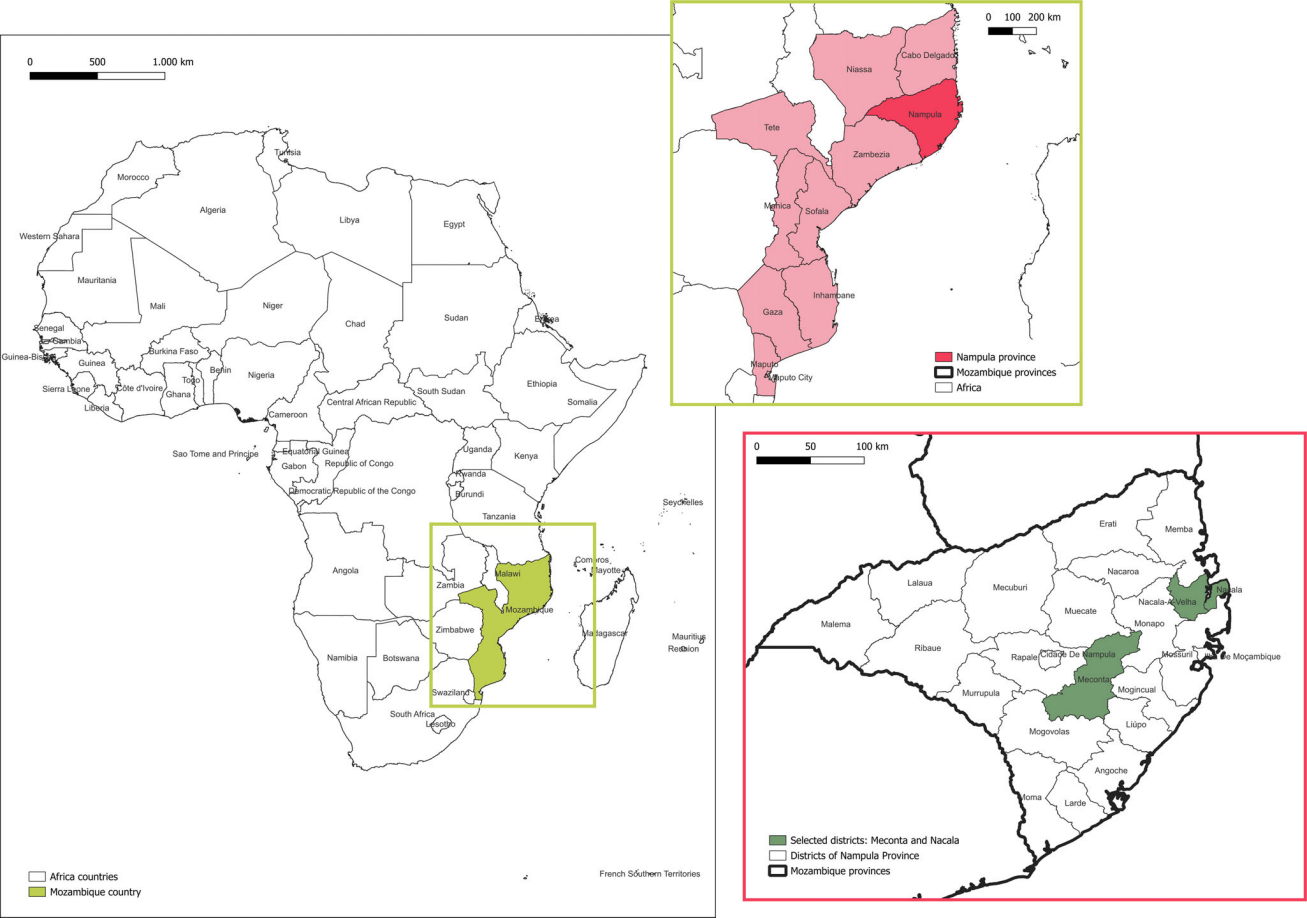
Localization of the selected study area in Mozambique, highlighting Nampula Province and the districts of Meconta and Nacala.

This study focuses on the Nampula Province in northern Mozambique, specifically on the districts of Meconta and Nacala, which are notable for their agricultural activities (Figure 1).

Meconta and Nacala were selected because they represent two complementary contexts, as well as they reflect the broader Mozambican setting. Meconta is a predominantly rural district with strong smallholder farming traditions, while Nacala combines rural agricultural communities with proximity to an important port city, facilitating agro‐processing and market access. Their selection reflects both empirical relevance and the potential for replicable interventions addressing the water–energy nexus. Nampula Province, covering an area of 79 010 km^2^, is the most populous province in Mozambique, with a population of approximately 5.76 million people. The province is characterized by a high poverty rate, with 65% of the population living in poverty, and a significant portion of the population experiencing food insecurity [21].

From a biophysical and environmental perspective, Nampula Province is characterized by a heterogeneous landscape susceptible to water erosion and soil degradation, intensified by deforestation and unsustainable farming. The decline in vegetative cover further reduces soil water retention and undermines local biodiversity.

The tropical subhumid climate of Nampula is marked by strong rainfall seasonality (October–April) and annual precipitation between 800 and 1200 mm. However, increasing climate variability has led to irregular rainfall patterns and more frequent extremes—such as droughts and intense storms—disrupting water balance, agroecosystem resilience, and food security.

From an agroecological standpoint, the regional economy is based on extensive, low‐input subsistence farming systems. Main crops include cassava, maize, groundnuts, legumes, and vegetables, cultivated on small plots using traditional methods with minimal mechanization and limited access to productive inputs or irrigation.

Access to basic services is highly uneven: urban centers such as Nacala have partial energy and water infrastructure, while most rural areas remain off‐grid. In these areas, electricity access is below 27%, and over 89% of energy use comes from traditional biomass (mainly firewood and charcoal), leading to deforestation, emissions, and health risks from indoor air pollution.

Water resources in the region are increasingly scarce and degraded. Many community supply systems are outdated, with limited capacity to meet demand. Poor planning and lack of integrated management are accelerating the depletion and contamination of surface and groundwater, worsened by inadequate treatment and sanitation, with serious impacts on health, agriculture, and ecosystems.

The study area was selected based on a set of criteria including: (i) agricultural relevance; (ii) presence of smallholder farming systems; (iii) availability of biomass residues; and (iv) critical challenges in water and energy access. Spatial and demographic data were retrieved from the National Institute of Statistics of Mozambique [22], while agronomic data were complemented by FAO [23] and IFAD [24] datasets, including information on crop distribution, farming systems, and productivity.

**FIGURE 2:**
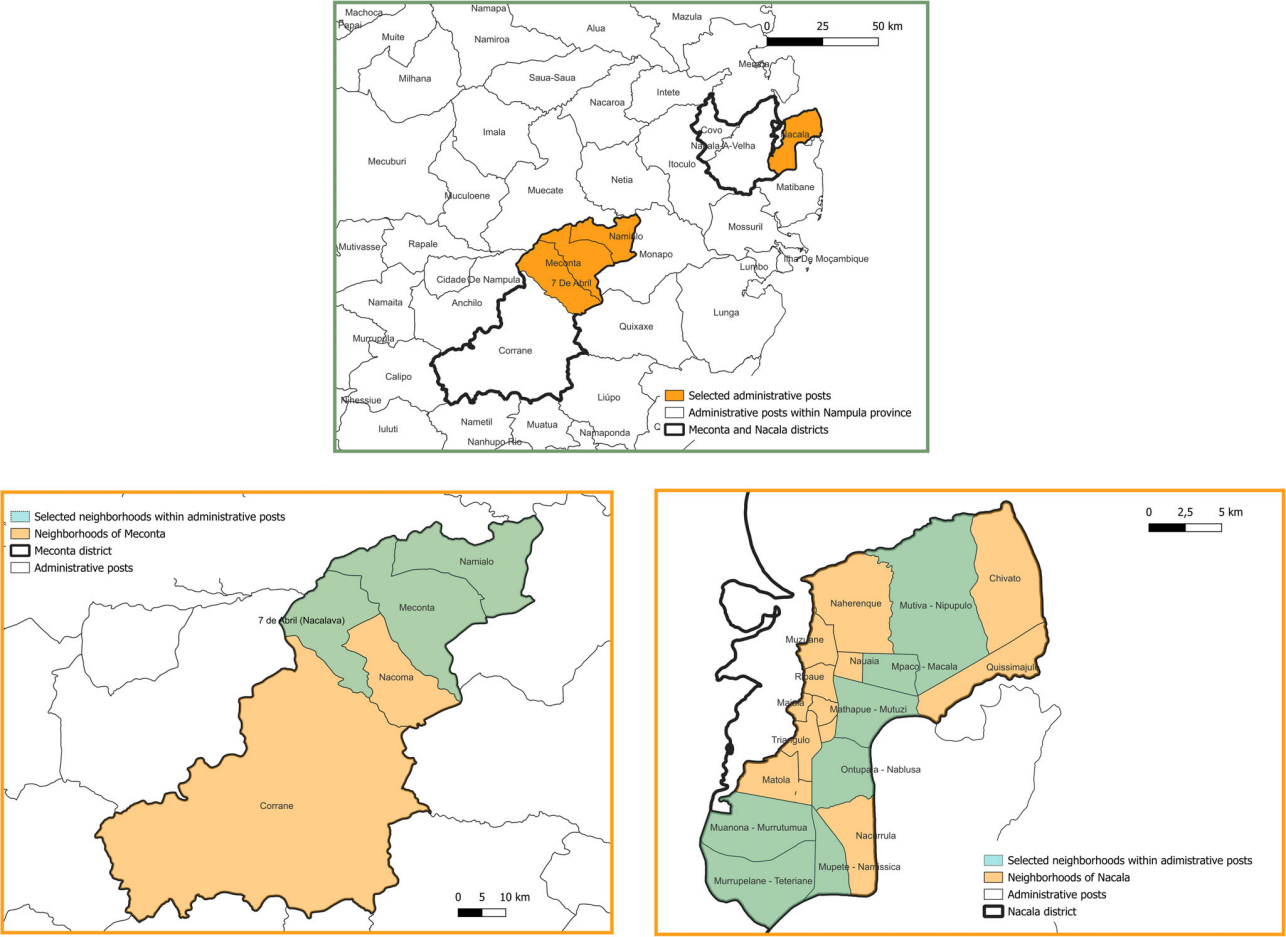
Selected neighborhoods within the Nacala and Meconta districts of Nampula Province, Mozambique.

In Meconta district, the administrative posts of Vila Sede de Meconta, 7 de Abril‐Nacavala, and Namialo were selected for analysis (Figure 2). Meconta has a population of 223 760, with a significant portion residing in rural areas.

In Nacala district, the study focused on the rural neighborhoods of Teterranea, Namissica, Nablusa, Mutiva, Mpaco, Mutuzi, and Murrutumua (Figure 2). These specific neighborhoods were chosen to capture the diversity of agricultural practices, water availability, and energy access within the districts. Nacala, also known as Nacala‐Porto, is a coastal city with a population of 287 536. However, the study excluded the urbanized coastal zone, concentrating instead on the surrounding agrarian communities, which are characterized by traditional, rainfed agriculture and limited infrastructure.

Crops cultivated in these areas, such as cassava, maize, and groundnuts, generate significant volumes of agricultural residues, including cassava peels, maize stalks, and groundnut shells. These by‐products are largely unutilized, despite their potential for transformation into bioenergy through processes such as anaerobic digestion or gasification [12]. According to recent studies on biomass valorization [12], these residues represent a potential feedstock for bioenergy production through anaerobic digestion or gasification, providing opportunities to reduce waste, generate energy, and mitigate deforestation.

Water and energy constraints were addressed through a nexus framework that considered the interdependence of resource flows in smallholder systems. Less than 3% of Mozambique's arable land is currently irrigated [25], which makes seasonal rainfall a critical variable for both biomass availability and food security. For example, the Meconta district experiences an average annual rainfall of approximately 857 mm, with the majority of precipitation occurring between the months of October and April, influencing both the availability of water and the agricultural calendar. At the same time, rural energy access remains below 10%, constraining post‐harvest processing, storage, and value addition [26].

The selected areas serve as a strategic case study for testing community‐driven, resource‐efficient solutions that address the interconnected challenges of water, energy, and food security. The approach aligns with the principles of the circular economy and the European Green Deal, aiming to convert agricultural waste into valuable resources and promote inclusive, sustainable rural development in Mozambique.

### 2.2 Data Preparation

The study adopted an integrated and participatory methodological approach aimed at analyzing critical issues related to the use of water and energy resources in the two selected districts of Nacala and Meconta, in Nampula Province, Mozambique. The investigation focused on the agricultural farms and agricultural processing industries, considered strategic for enhancing the resilience of local socioecological systems. The choice of combining GIS‐based spatial analysis with participatory surveys was driven by the need to simultaneously capture (i) the spatial distribution and magnitude of agricultural residues and water resources, (ii) the local practices and socioeconomic constraints, and (iii) the opportunities for community‐driven interventions. Spatial analysis provides precise, reproducible quantitative insights, while participatory surveys ensure context‐specific, socially grounded understanding. The methodological design was inspired by the principles of participatory action research and collaborative territorial planning [27, 28], promoting the active and continuous engagement of local stakeholders in the co‐generation of knowledge and in identifying intervention priorities.

In line with the systemic and multidisciplinary approach recommended in the literature on integrated natural resource management [29, 30], the methodological process was structured into three interlinked phases: i) institutional collaboration and secondary data collection; ii) exploratory territorial analysis and stakeholders engagement; and iii) field surveys and empirical data collection.

Initially, a collaborative network with local institutions was established, which was considered a prerequisite for the effective implementation of field research. The initiative was formally presented to the *Serviço Distrital de Actividades Econômicas* (SDAE) and the *Serviço Distrital de Planificação e Infra‐estrutura* (SDPI), which provided institutional support, facilitated access to existing data, and accompanied the research team throughout its territorial activities. This phase contributed to strengthening the social legitimacy of the process and to activating institutional cooperation essential for the effectiveness of subsequent actions [31].

Concurrently, an exploratory territorial analysis was conducted with the objective of identifying priority areas for intervention. This analysis combined the consultation of secondary data provided by local authorities, a critical review of available environmental and planning documents, and a desk‐based analysis of previous experiences in the districts under study. These efforts were complemented by participatory meetings with community representatives, aimed at collecting qualitative information and direct perceptions of major issues related to access and management of water and energy resources. The approach aimed at valorizing local knowledge as a tool for community empowerment as well as an essential component for the contextualized analysis of territorial dynamics [32, 33].

Following this initial phase, the eleven neighborhoods, belonging to the two districts of the Meconta and Nacala districts, were selected as priorities for the investigation. These areas became the focus of the subsequent data collection phase. Based on the preliminary information acquired and in order to ensure a rigorous, systematic, and culturally sensitive methodological approach, two structured questionnaires were developed and subsequently validated to address specific characteristics of the target groups: 1) farmers and 2) agricultural processing industries.

For both questionnaires, clear inclusion and exclusion criteria were applied to ensure methodological rigor. Farmers were eligible if they cultivated at least one of the major local crops (e.g., cassava, maize, or groundnuts), owned or managed land in the selected neighborhoods, and were directly involved in residue management and water/energy use. Farmers who were seasonal workers without decision‐making roles or who did not reside in the study area were excluded. For agro‐processing industries, only enterprises formally or informally operating within the districts and processing local agricultural products were included, while businesses unrelated to food production or located outside the study boundaries were excluded. The development of these data collection tools followed a participatory and context‐sensitive design process, considering the specificities of the local rural context, prevailing agricultural practices, and the socioeconomic conditions of the target communities. In particular, the design of the questionnaires adhered to an internal logic of coherence between the research's knowledge objectives and the analytical dimensions to be explored (natural resources, production practices, access to water and energy services, and technical and managerial capacities of local actors). Variables were selected based for their empirical relevance to the water‐energy‐agriculture nexus [12, 23, 24], and included crop type, cultivated area, biomass volume, water consumption, and energy use. These variables also reflect determinants identified in prior literature as critical for designing community‐scale bioenergy and irrigation interventions.

The content of the two questionnaires was developed involving:

- The analysis of available secondary data (environmental reports, local development plans, and prior studies conducted in the target districts);
- Consultations with local and institutional stakeholders (including SDAE and SDPI);
- The empirical knowledge of the territory acquired through exploratory field missions and community meetings.

The questionnaires were organized into thematically coherent and user‐friendly sections and were designed to be administered through guided interviews. This approach aimed to minimize cognitive and linguistic biases while fostering more meaningful, inclusive, and effective engagement between interviewers and participants. In this regard, special attention was paid to using clear yet precise language, which was later translated into Portuguese and, where necessary, supported by oral translation into Emakhuwa (Macua), the predominant local language in Nampula Province.

For the fieldwork phase, the questionnaires were digitized using KoboToolbox, an open‐source platform widely employed in humanitarian and environmental research contexts for data collection in low‐infrastructure geographic areas [34, 35]. The choice of this tool allowed the creation of a flexible, interoperable, and field‐adapted data collection system, thanks to its offline capabilities and subsequent synchronization functionality.

Prior to the official launch of the data collection campaign, an intensive training session was held for the field operators responsible for administering the questionnaires. The training had a dual purpose: first, to equip participants with the necessary technical skills to effectively use the KoboToolbox platform (data entry, error management, data synchronization); second, to share methodological guidelines for conducting effective interviews, with particular attention to intercultural communication and the use of the local language. This latter aspect proved essential for ensuring respectful, clear, and culturally appropriate communication with participants, thereby minimizing language barriers and potential data collection biases [36].

All data collection procedures adhered to strict ethical standards. Prior to participation, farmers and enterprise representatives were informed about the objectives of the research and the voluntary nature of their involvement. Informed consent was obtained verbally or in writing, depending on literacy levels, and participants were assured of confidentiality and anonymity. Personal identifiers were not stored, and all data were kept securely in encrypted files accessible only to the research team. Ethical approval for this study was obtained from a local NGO, in accordance with national and international research ethics guidelines. A pilot testing (pre‐test) phase was conducted in selected sample locations before the full rollout of data collection. This phase aimed to validate the effectiveness of the digital tools in the field, assess respondent comprehension of the questions, and identify any technical or procedural weaknesses. During the pre‐test, several limitations emerged related to poor network coverage in remote rural areas, which affected real‐time updates and data synchronization. To address these issues, the offline functionality of the platform was activated, allowing for uninterrupted data collection and ensuring no data loss.

Proactive management of the issues encountered during the pre‐test contributed to strengthening the operational robustness of the data collection system and improving the field operators’ capacity to address the logistical and technological challenges typical of rural and fragile contexts such as those observed in the selected study areas (e.g., districts of Nacala and Meconta).

The first questionnaire, designed for farmers and agricultural enterprises, aimed to collect detailed information on cultivated crops, the use of biomass derived from agricultural residues, water consumption and management for irrigation, irrigation practices, and energy requirements (Questionnaire S1). The second questionnaire, targeting agro‐food processing enterprises, focused on energy consumption, resource management practices (including water, energy, and raw materials), and the main operational challenges within the local production context (Questionnaire S2).

Once the framework was defined and the methodological tools were tested, an intensive field data collection campaign was launched in the districts of Nacala and Meconta, with the objective of obtaining a detailed and reliable information base on the local agricultural and agricultural processing sectors. The fieldwork was conducted between September 2024 and February 2025 by four trained operators, organized into two territorial units: two assigned to the Nacala district and two to the Meconta district. This organization ensured comprehensive territorial coverage and enhanced responsiveness to the specific contexts of each area, thereby improving both data collection efficiency and the quality of the information gathered.

The adopted methodological approach enabled the collection of reliable and consistent quantitative and qualitative data on the environmental, water, energy, and agricultural conditions of the study areas, while also fostering greater local community engagement in the research process. Such active participation is considered an essential component of investigations in rural settings, contributing to higher ecological validity of the data and strengthening local ownership of the results [27, 29].

Nonetheless, several operational challenges emerged during the data collection phase, affecting both the timing and territorial coverage of field activities. Chief among them were extreme weather conditions: the passage of tropical cyclones CHIDO (December 2024) and DIKELED (January 2025) caused severe damage to road infrastructure, making access to many rural communities difficult. These were compounded by a context of political instability following the presidential elections in October 2024, which led to episodes of protest and social unrest, limiting operator mobility and significantly slowing down operations [37].

Additional logistical complications stemmed from the vast distances between data collection points and the low accessibility of many rural areas, especially during the rainy season (e.g., from November to March). At the outset, a degree of reluctance was also observed among farmers and enterprises to participate in the survey, due to unfamiliarity with research activities and concerns about the use of collected data. This resistance was progressively overcome through community information sessions aimed at clearly explaining the project objectives and building trust in the research team [38].

The most critical period occurred in December 2024, during which a marked drop in interviews was recorded due to the combined impact of extreme weather events and political‐social tensions. Despite these limitations, the adaptive strategy and the use of the platform's offline mode ensured methodological coherence and data integrity throughout the process.

### 2.3 Data Analysis

Prior to conducting spatial analyses and developing the GIS methodology, a comprehensive geodatabase was established by integrating existing data with field survey information, providing a robust and context‐specific foundation for subsequent territorial assessments. This database served as the foundation for a GIS‐based spatial model designed to identify priority zones for agricultural waste valorization and water management interventions. Then, the methodology involved the following key steps:

1. collection of local institutional data (from SDAE, SDPI, Ministry of Public Works of Mozambique) concerning soil, agriculture, water and energy networks, urban and erosion‐risk areas, and water supply points;
2. GIS mapping of the study area with preliminary maps produced for selected rural neighborhoods within the Nacala and Meconta districts;
3. georeferencing of survey data by localizing interviewed farms and agro‐industries using GPS coordinates for spatial visualization;
4. quantitative assessment of agricultural resources through indicators such as cultivated area, crop types, production volumes and residues, energy consumption, freshwater use for irrigation, and livestock numbers with associated waste;
5. GIS‐based thematic mapping to highlight spatial distributions of the collected data;
6. creation of weighted district‐level heat maps illustrating the distribution and intensity of agricultural residues, cultivated areas, production, and irrigation water use;
7. identification of strategic hotspots within the water–energy nexus, prioritizing zones with high concentrations of agricultural residues for potential bioenergy technology implementation to enhance energy access;
8. identification of critical water management areas characterized by high freshwater irrigation demand, targeted for the development of sustainable management guidelines within the water–energy nexus framework.

The spatial analyses underpinning the GIS‐based model were conducted using QGIS (v.3.36.3), a widely recognized open‐source tool for geospatial analysis [39, 40, 41, 42, 43, 44]. Its robust functions in data integration, management, and visualization made it particularly suitable for application in rural Mozambique. The model, based on a cartographic approach, supports the spatial assessment of territorial dynamics to guide planning in the water‐energy nexus. Within this framework, the analysis focused on identifying two categories of strategic zones. First, potential areas for the sustainable valorization of agricultural waste and by‐products were identified based on the highest availability of biomass resources, adopting the heatmap plugin available in QGIS. These areas were prioritized for the possible siting of valorization technology installations, with considerations for minimizing transport distances, reducing logistical and environmental burdens, and lowering associated greenhouse gas emissions. Second, always adopting heatmap plugin, critical zones with the highest levels of freshwater use for irrigation were delineated, highlighting them as priority areas for water management interventions within the nexus framework. The raster merge tool available in QGIS was used to identify the critical areas and the opportunity hotspots within the water–energy nexus.

The empirical approach emphasizes transparency and replicability. Each method (e.g., survey, GIS mapping, weighted overlay) is justified by its contribution to answering specific research questions: spatial allocation of biomass, identification of water‐energy nexus vulnerabilities, and prioritization of sustainable interventions. By explicitly linking the methods to the research objectives, the study ensures that empirical results are interpretable, actionable, and grounded in both technical and socioeconomic realities.

## 3 Results and Discussion

### 3.1 Data Analysis

The field survey yielded a broad and representative empirical sample of the agricultural and agro‐industrial realities in the districts of Nacala and Meconta. In Nacala, 631 farmers were interviewed out of a total of 1450 registered in the target neighborhoods (43%), while all registered processing enterprises (2 out of 2) were successfully reached, yielding 100% coverage (Table 1). In Meconta, 60 farmers were surveyed—exceeding the official number of registered producers (54)—due to the intentional inclusion of six previously undocumented agricultural enterprises, identified through local knowledge and considered operationally relevant. Similarly, 26 processing enterprises were surveyed in Meconta, compared to 24 officially recorded, indicating the widespread presence of informal economic actors not captured by institutional databases—a phenomenon commonly observed in rural sub‐Saharan contexts [45].

**TABLE 1:** Interviewed actors within Nacala and Meconta districts.

	Nacala			Meconta		
	Recorded	Interviewed	% Interviewed	Recorded	Interviewed	% Interviewed
Farmers	1450	631	43%	54	60	111%
Agri‐food processing industries	2	2	100%	24	26	108%

The spatial distribution of the sample revealed a marked concentration of agricultural activity in the neighborhoods of Mutuzi and Namissica, which together accounted for over 32% of all interviews. These were, followed by Nablusa, Teterranea, Mpaco, and Mutiva. In contrast, peripheral zones and the municipal center of Meconta showed lower levels of representation (Figure S1). This pattern highlights a higher agricultural density in semi‐peri‐urban areas, where farming practices tend to be more established and structured.

From a socioproductive perspective, the surveyed population was predominately composed of individual farmers who made up 84.42% of the sample. Organized forms of agricultural activity were minimally represented (Figure S2): informal associations accounted for 9.38%, while structured agricultural enterprises comprised less than 5%. Notably only a single cooperative was identified across the entire sample, underscoring the institutional and cultural barriers that hinder the development of cooperative models. Despite this, existing research indicates that associative structures are generally more effective in promoting sustainable practices, improving access to credit, and enabling the collective valorization of agricultural production [46].

Agricultural practices among the surveyed farmers were predominantly oriented toward monocropping, with over 70% of respondents cultivating a single crop. As already mentioned, the primary crops included maize, cassava, soybeans, and millet. Only 27% of farmers reported engaging in polyculture, and fewer than 34% employed intercropping techniques. This indicates limited uptake of agroecological practices, despite substantial evidence that intercropping—particularly cereal‐legume systems—can enhance soil fertility and bolster resilience to climate‐related stresses [47, 48]. Agriculture remains poorly integrated with livestock rearing: only 3% of farmers (21 out of 691) practiced mixed farming, primarily involving small‐scale goat and poultry rearing. The overwhelming majority (668 cases) focused exclusively on crop production. The limited prevalence of integrated farming systems in Nacala and Meconta can be attributed to several factors, including limited land availability, lack of technical knowledge, absence of veterinary support, and restricted access to start‐up capital.

Farmers in both districts typically follow a dual cropping cycle per year, taking advantage of both the rainy and dry seasons. However, the continuity and productivity of these cycles are significantly hindered by limited water availability—a critical structural constraint within the local agricultural system. Data revealed a concerning gap in irrigation infrastructures: 61.76% of farmers (428 out of 691) rely entirely on rainfall, which is both highly variable and unreliable (Figure S3). Only 34.92% (242 respondents) reported using any form of irrigation, and even among these, irrigation is almost exclusively manual. This leads to low water‐use efficiency and high labor demands, further limiting the potential for year‐round agricultural productivity.

The adoption of advanced irrigation technologies remains extremely limited: only five farmers reported using pressurized or drip irrigation systems, and just one association in Nacala owns a solar‐powered irrigation pump (Figure S4). This scarcity of modern irrigation infrastructures significantly restricts the productivity potential of local agriculture and exacerbates its vulnerability to climate‐induced risks. As highlighted by FAO (2021) [49] and the IPCC (2022) [50], the implementation of appropriate irrigation technologies—such as renewable‐energy‐powered micro‐irrigation systems—represents a critical strategy to enhance agriculture resilience, reduce rainfall dependence, and optimize water use in vulnerable regions such as northern Mozambique.

Similarly, mechanization levels are remarkably low: approximately 98% of farmers carry out land preparation operations exclusively using manual tools, with only four interviewed farmers reporting the use of tractors or other mechanical equipment (Figure S5). This widespread lack of mechanization limits agricultural productivity and places a significant physical burden on farmers—particularly women, who represent a large proportion of the rural labor force. In line with FAO (2016) guidelines [51], the introduction of lightweight, context‐appropriate mechanization can enhance agricultural efficiency, reduce manual labor, and support sustainable, climate‐resilient practices.

Post‐harvest practices indicate a production system largely focused on immediate sales, with minimal investment in storage or processing infrastructure. Nearly half of the farmers (48.77%) sell their harvests immediately after collection, while 46.18% primarily retain produce for household consumption, reflecting a subsistence‐oriented approach rather than market integration (Figure S6). Only a small fraction (6.93%) engages in primary processing, which is typically rudimentary and often lacks adherence to quality standards.

Agricultural residue management also shows considerable potential for improvement. Although a small portion of residues is repurposed as compost or livestock feed, a significant share is routinely burned in the fields or discarded. This practice leads to a loss of valuable organic matter and contributes to the release of climate‐forcing pollutants, particularly CO_2_ and fine particulate matter.

In the district of Nacala, two agro‐processing industries were interviewed, representing 100% of the officially registered businesses within the project's intervention areas (Teterranea and Mutiva). The low number reflects the exclusion of Nacala's industrial zone—where most such industries are concentrated—from the project scope. In Meconta, 26 interviews were conducted compared to 24 registered enterprises, revealing the presence of informal operators involved in agricultural processing. This informality, common in Mozambique and across much of Africa, often results from weak regulatory enforcement and barriers to formal business registration.

Regarding the territorial distribution of interviews within Meconta district, the majority took placed in the municipality of Meconta (16 interviews, or 57.14%), followed by 7 Abril‐Nacavala (9 interviews, or 32.14%). In the neighborhoods of Teterranea, Namialo, and Mutuzi each accounted for one interview (3.57%), reflecting the relatively sparse presence of agro‐processing industries in these areas (Figure S7).

The surveyed agro‐processing industries reflect a predominantly private and informal productive structure, with a marked absence of cooperatives. The most common equipment includes maize and cassava mills, cashew and peanut shellers, and rice and cereal dehuskers. However, the production of fortified flours—recognized for their role in combating child malnutrition [52]—is virtually absent due to limited access to blending technologies and a general lack of awareness about nutritional benefits of such products.

An essential aspect to consider is the type of clientele served by agro‐processing centers. Unlike industrialized markets, where large agricultural and agribusiness companies dominate the value chain, in the districts of Nacala and Meconta the primary clients of these centers are women from local communities. These women bring their sacks of cereals—mainly maize, sorghum, and cassava—to mills and processing units for transformation into food products intended for household consumption and family sustenance. This pattern reflects a small‐scale, community‐oriented processing model typical of rural African contexts, where agricultural value chains remain decentralized and minimally integrated with commercial enterprises.

The management of residues and by‐products resulting from agro‐processing activities remains a challenge, with limited dissemination of valorization practices. Although in some cases residues are repurposed—for instance, as boiler fuel or animal feed when quality allows—most by‐products are discarded without any productive use. In other instances, residues are fermented to produce traditional alcoholic beverages, which are popular in rural areas due to their low cost. However, these practices are largely informal and lack broader sustainability or economic optimization.

An innovative exception was identified in Meconta, where microenterprises have begun processing cashew apples into juice. This emerging activity stands apart from the well‐established cashew nut export chain. The cashew apple, which constitutes around 90% of the total biomass of the plant and is typically discarded during nut processing, is rich in vitamin C and holds significant nutritional and commercial potential. While the juice is sold locally, production remains small‐scale due to critical infrastructure gaps—including the absence of cold storage, pasteurization equipment, and reliable transport systems. These constraints severely limit shelf life, restrict market reach, and inhibit the scalability of operations.

From an energy perspective, agro‐processing activities in both districts reveal an almost complete lack of energy integration. Survey data show that 97.98% of enterprises operate without any form of energy input (Figure S8). Only 1.3% of operator's report using energy—primarily through outdated, inefficient equipment such as mills and shellers powered by unstable grid connections or fossil fuels (diesel, paraffin). Where energy is used, it often results in high operational costs and increased environmental impacts due to CO_2_ emissions and reliance on non‐renewable resources. In a few isolated cases, biomass‐ or wood‐fired boilers fueled partly by agro‐industrial residues were reported, suggesting a marginal but potentially scalable opportunity for circular energy practices and decentralized self‐generation.

### 3.2 KoboToolbox Results Elaboration

Following the completion of the field data collection phase, raw data were exported from the KoboToolbox platform and underwent a structured process of data cleaning, coding, and preliminary statistical analysis. This phase aimed to ensure consistency, accuracy, and analytical readiness. The processing combined the use of quantitative analysis tools, primarily Excel, with GIS platform, enabling a multidimensional interpretation of the data across spatial, socioeconomic, and agro‐environmental dimensions.

The analytical approach was designed to achieve three core objectives:

i. the spatial identification of critical issues related to access to water and energy, with a focus on pinpointing areas characterized by infrastructural vulnerability;
ii. the reconstruction of land use patterns and the spatial distribution of agricultural activities, in order to highlight areas with strategic potential for the adoption of sustainable technologies;
iii the generation of thematic maps to support evidence‐based territorial planning, resource allocation, and the formulation of rural development policies.

All activities were carried out in accordance with rigorous methodological standards and principles of scientific replicability. Particular attention was paid to maintaining coherence between the structure of the questionnaire, the coding schema, and the variables processed during the analysis. The integration of descriptive statistics with spatial mapping allowed for the quantification of observed dynamics and their contextualization within the specific territorial realities of Meconta and Nacala. This approach provided a nuanced understanding of the interconnections within the water–energy–agriculture (WEA) nexus.

Furthermore, during the preliminary phase, the data were organized into thematic categories, such as cultivated area and water use, crop typology, livestock distribution, and agricultural production, as shown in the following sections. This thematic segmentation facilitated their incorporation into GIS workflows, supporting detailed, spatially explicit interpretations and helping to identify both critical challenges and development opportunities within the study area.

**FIGURE 3:**
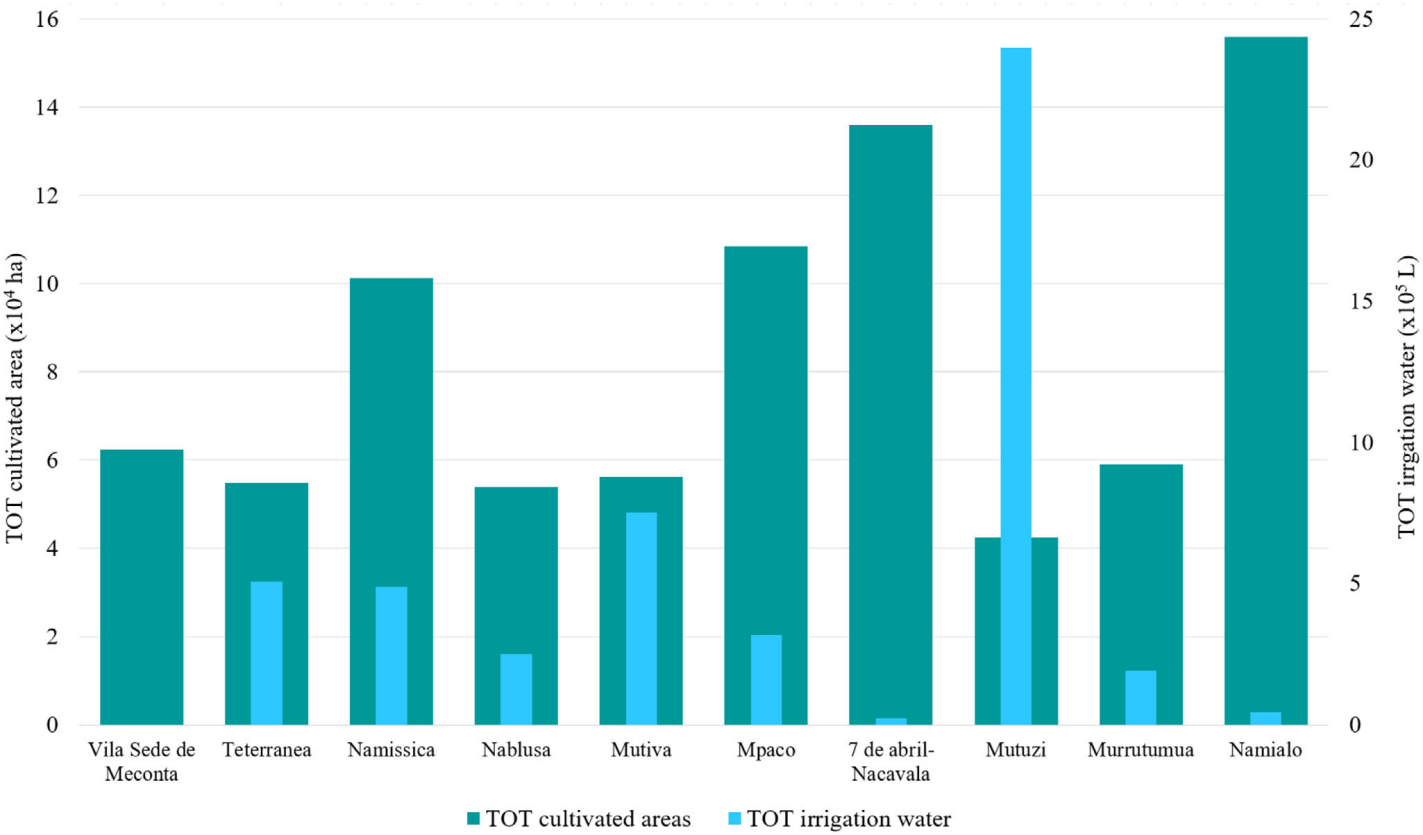
Cultivated area and annual irrigation water use across Meconta and Nacala districts.

Field data analysis indicates that the total cultivated area across the Meconta and Nacala districts is approximately 831 000 ha, with Meconta accounting for 42.7% and Nacala for 57.3%. The most intensively cultivated neighborhoods are Namialo and 7 de Abril‐Nacavala in Meconta, and Mpaco and Namissica in Nacala (Figure 3). The total annual irrigation water use across both districts amounts to approximately 4.98 million liters. However, a significant imbalance is observed: Nacala consumes 98.6% of total water use, despite its cultivated area being only moderately larger than Meconta's. This discrepancy suggests that irrigation water consumption is more strongly influenced by crop types, irrigation practices, and local climatic conditions than by cultivated area alone. Neighborhoods such as Teterranea and Mutiva show irrigation volumes closely aligned with their cultivated area, indicating relatively stable water demand per hectare. Conversely, Mutuzi exhibits disproportionately high‐water consumption, likely due to water‐intensive crops or inefficient irrigation practices. Namialo, on the other hand, exhibits notably low irrigation water use despite a large cultivated area—potentially reflecting more efficient irrigation systems or the predominance of low‐water‐demand crops.

**FIGURE 4:**
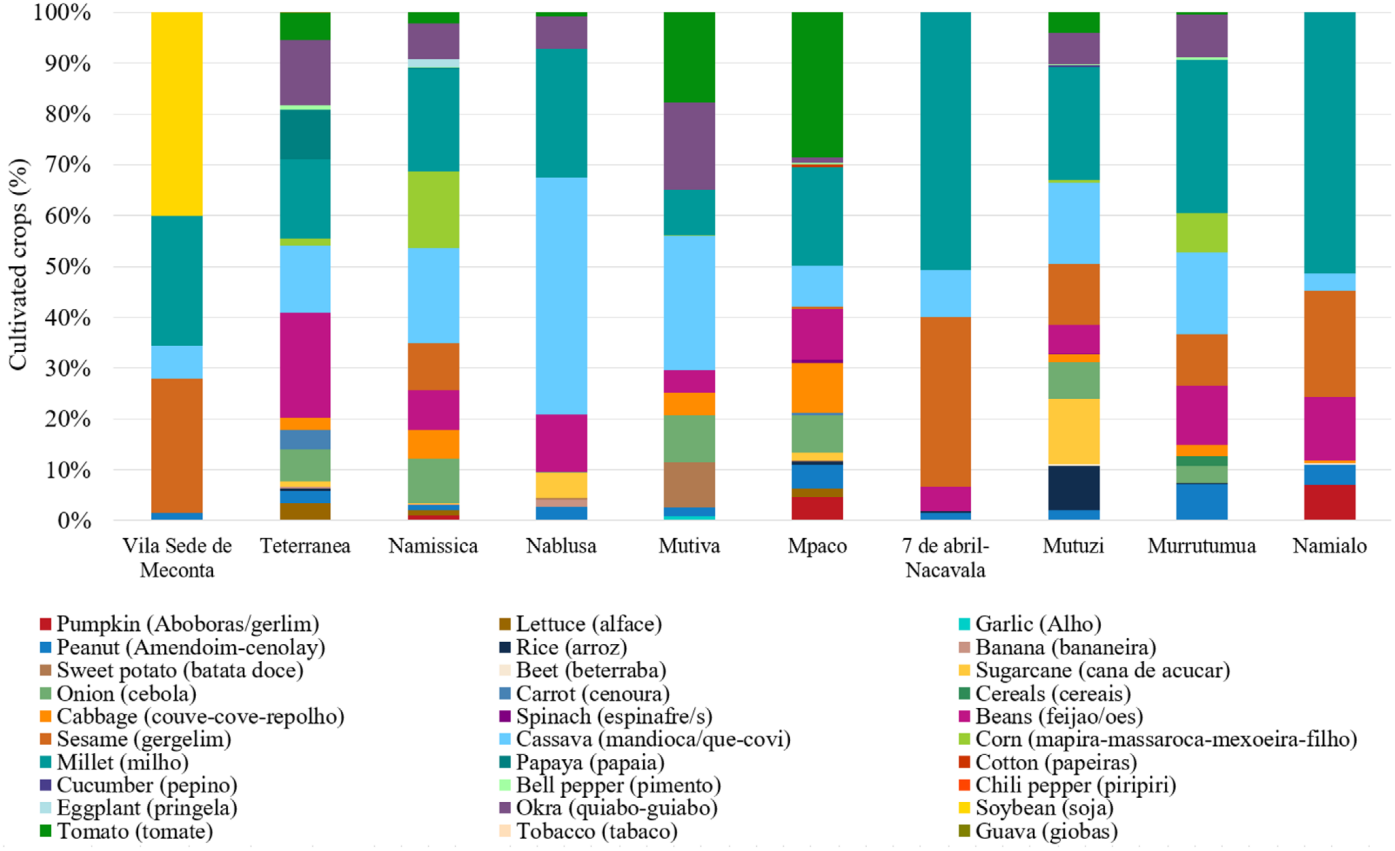
Cultivated crops across Meconta and Nacala districts.

As shown in Figure 4, among the crops cultivated in the Meconta and Nacala districts, millet is the most widely cultivated, covering on average 26.9% of the total agricultural land. Its prevalence is especially evident in neighborhoods such us 7 de Abril‐Nacavala and Namialo, where it accounts for over half of the cultivated area. Cassava follows with 16.4%, underscoring its role as a calorie‐dense, resilient staple well‐suited to diverse agroecological conditions. Sesame and beans, although more moderately represented (11.3% and 8.9%, respectively), are consistently found throughout the region, suggesting their importance both as dietary staples and cash crops. Interestingly, some crops display highly localized concentration. Interestingly, there are also cases where a specific crop is intensely cultivated in only one area while being almost entirely absent elsewhere. A clear example is seen in Vila Sede de Meconta, where a single crop covers 40% of the local cultivated area but contributes just around 4% when averaged over the entire region. These localized patterns highlight how agricultural choices can vary sharply even within a relatively compact territory, influenced by factors such as soil conditions, market access, tradition, or local expertise. The result is a heterogeneous agricultural landscape, where certain crops dominate widely, while others remain confined to specific areas.

**FIGURE 5:**
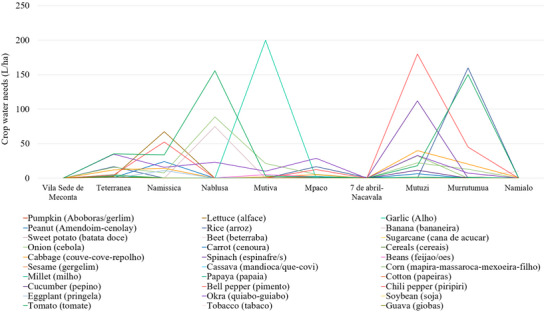
Crop water needs across Meconta and Nacala districts.

Analysis of crop‐specific irrigation requirements reveals substantial variability. In Mutiva, garlic exhibits the highest water demand, exceeding 250 L/ha, well above the average (Figure 5). Similarly, pumpkin and lettuce in Mutuzi reach peaks of approximately 180 L/ha and 120 L/ha respectively, marking this neighborhood as a hotspot of water‐intensive cultivation. Peanuts also show high variability, with water needs rising to around 160 L/ha in Nablusa, while remaining under 50 L/ha in other areas. In contrast, cassava and millet consistently require less than 20 L/ha across all neighborhoods, confirming their status as low‐input, drought‐tolerant crops suitable for regions with limited water resources. Beans, cabbage, and sweet potato fall in a moderate irrigation range of 30–70 L/ha, with relatively stable water needs.

Geographically, Mutiva, Mutuzi, and Murrutumua emerge as the areas with the highest cumulative water demands, each showing multiple crops with values exceeding 100 L/ha, suggesting either higher irrigation potential or a greater prevalence of water‐intensive crop choices. On the opposite end, neighborhoods such as Namialo and Vila Sede de Meconta show uniformly low values, with most crops requiring less than 30 L/ha, indicating a more extensive or rainfed agricultural approach. Overall, the neighbor of Mutuzi exhibits the highest total water demand, exceeding 450 L/ha, mainly due to significant contributions from pumpkin, lettuce, banana, and sesame cultivation. Murrutumua follows with approximately 390 L/ha, with rice and sesame being the dominant water‐consuming crops. Nablusa ranks third with around 340 L/ha, showing a high demand particularly for corn, sugarcane, and sesame cultivations. Namissica and Mutiva display moderate water needs, with total values of about 240 and 230 L/ha, respectively; Namissica's demand is more evenly distributed among several crops, while Mutiva's is dominated by garlic. Teterranea shows a lower but notable demand of roughly 140 L/ha. Mpaco and Namialo show relatively low water requirements, around 90 and 20 L/ha, respectively. Notably, 7 de Abril‐Nacavala shows a negligible water demand, barely reaching above zero.

**FIGURE 6:**
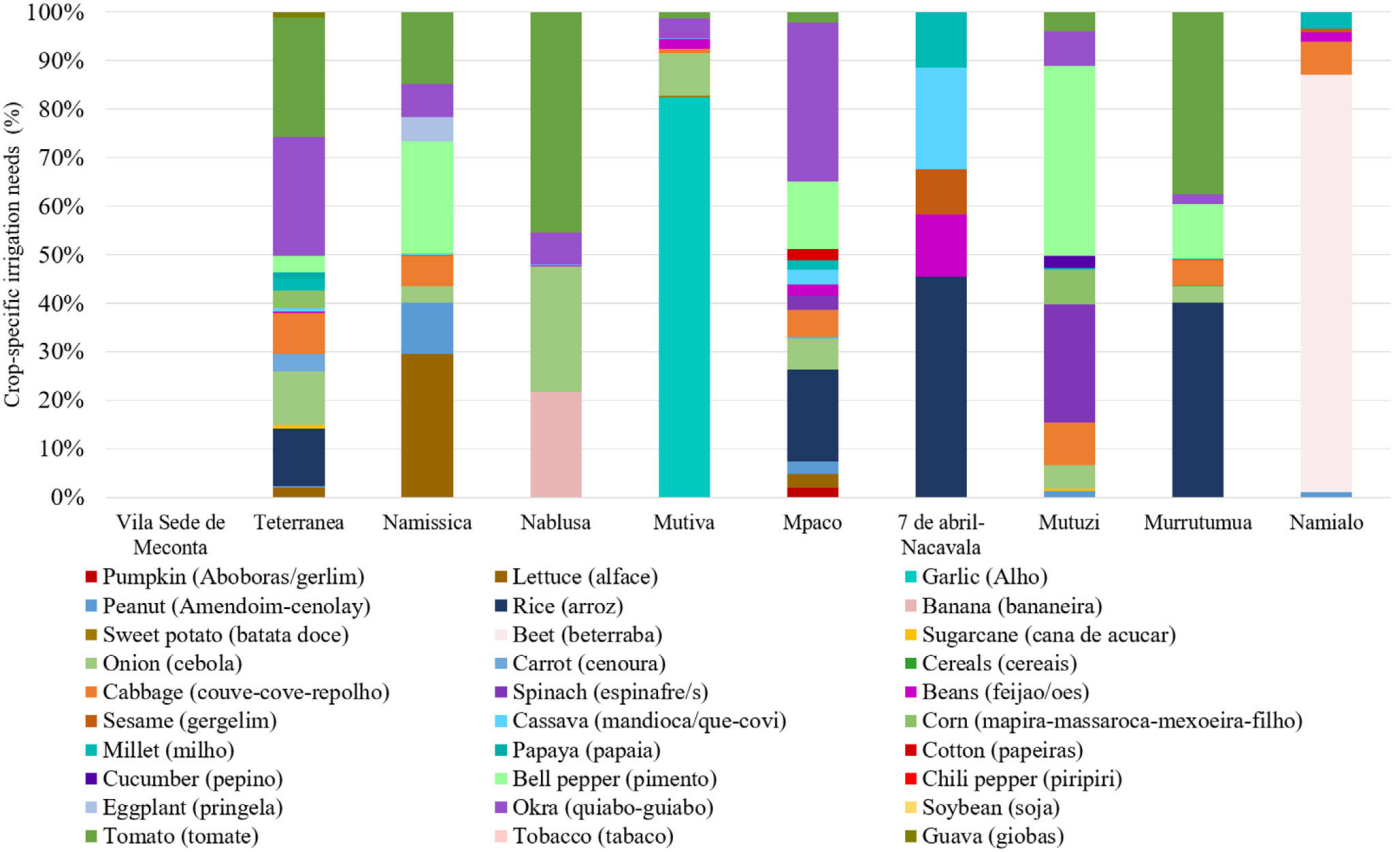
Crop‐specific irrigation needs across Meconta and Nacala districts.

The distribution of crop‐specific irrigation needs across the neighborhoods reveals significant heterogeneity in agricultural water dependency (Figure 6). In Mutiva, garlic alone accounts for approximately 82% of total water demand, marking it as a highly water‐intensive cropping system. Similarly, in Namialo, beet represents about 88% of local irrigation needs, underscoring a heavy reliance on a single crop. These monocultural trends can heighten vulnerability to water shortages, particularly in regions with limited irrigation infrastructures. Conversely, neighborhoods such as Tererranea and Namissica exhibit more diversified irrigation profiles. In Tererranea, spinach and peanut are the primary water‐consuming crops, accounting for approximately 25% and 23% of total irrigation demand, respectively. Additional contributions from cabbage, onion, and rice—collectively making up 10–12%—further highlight a well‐distributed water use pattern across several crops. Namissica also exhibits a broad distribution, with lettuce (29%), pumpkin (19%), and peanut (11%) forming the core of water consumption. This composition suggests a deliberate mix of crops with varied but moderate irrigation needs, contributing to a more resilient agricultural system. Nablusa presents a semi‐diversified structure: with banana contributing about 22%, onion 19%, and peanut 23%, reflecting a moderate concentration in a few crops but with some level of diversification. Mpaco is characterized by a substantial share of spinach (33%) and cabbage (27%), together accounting for over 60% of total irrigation needs. 7 de Abril‐Nacavala and Murrutumua both show a strong presence of cassava, which comprises 45% and 39%, respectively, of local irrigation demand. Although cabbage and garlic are also present, their shares are comparatively minor. These neighborhoods appear to emphasize moderately water‐demanding crops within a less diversified framework. Mutuzi, despite having a smaller cultivated area, shows a relatively balanced irrigation profile led by lettuce (26%), pumpkin (21%), and banana (11%). This distribution suggests a well‐integrated mix of vegetable crops with moderate irrigation needs, offering a degree of buffer against water stress or crop failure.

**FIGURE 7:**
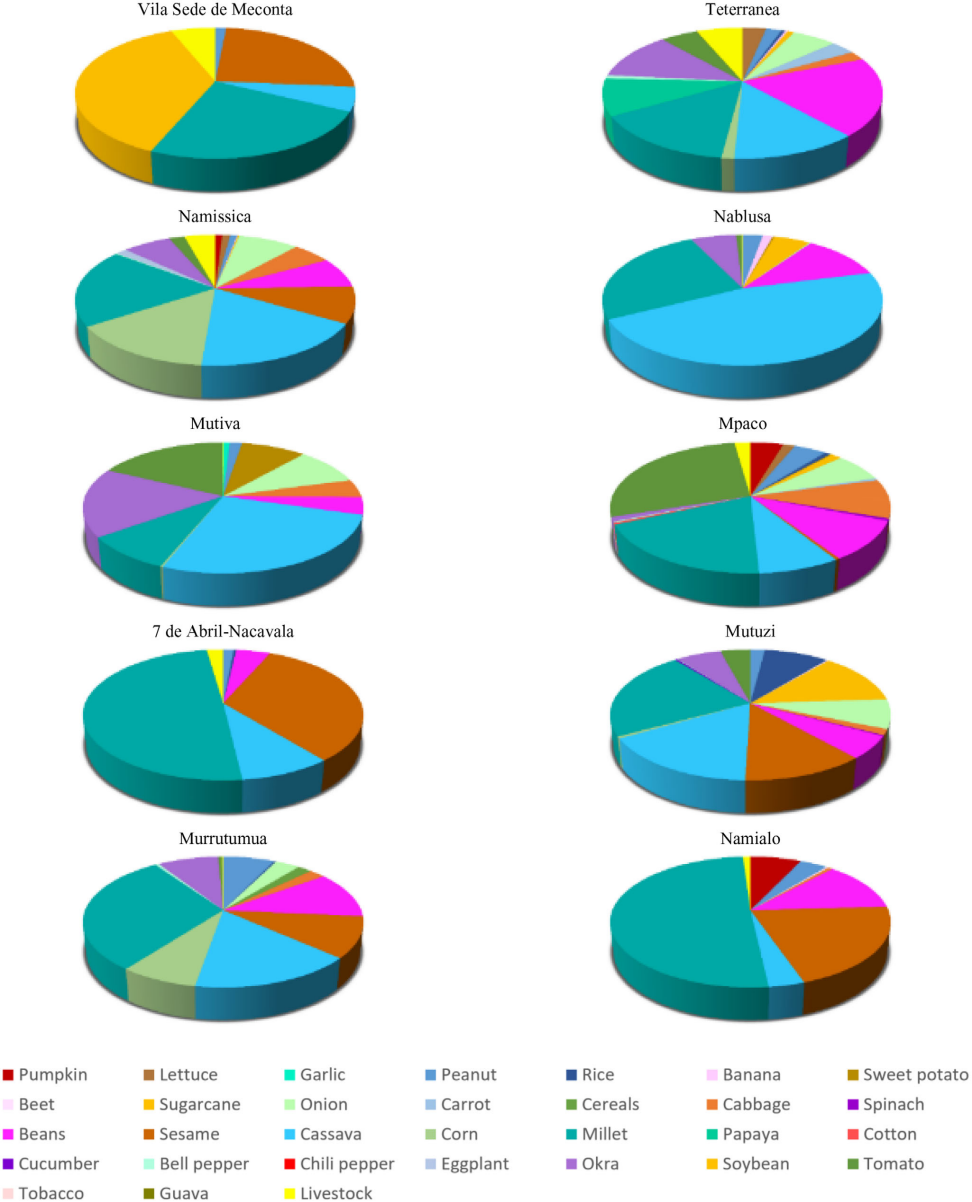
Land use distribution across Meconta and Nacala districts. The data are reported as the percentage on the total area, defined as the sum of land allocated to crops and livestock.

Data on land use distribution (Figure 7) reveal that Namialo is covered by the largest production—defined as the sum of land allocated to both crops and livestock—spanning 157 501.5 ha. The landscape is heavily dominated by millet, which accounts for over half of the land use (50.73%), followed by sesame (20.63%) and beans (12.38%), indicating a clear orientation toward drought‐resistant staple crops. Livestock, however, occupies only 1.02% of the area, reinforcing a crop‐dominated land use structure. In comparison, 7 de Abril‐Nacavala with 139 120 ha also shows a major emphasis on millet (49.60%) and sesame (32.71%), but with a modest presence of cassava (8.99%) and a slightly greater share of land used for livestock (2.24%), suggesting a modest integration of animal production into the system. These patterns likely reflect agroecological conditions suited to resilient, low‐input crops, possibly linked to semi‐arid zones.

Other areas exhibit more diversified use of land. For instance, Namissica with 105 713 ha, displays a more balanced distribution among cassava (17.79%), maize (14.44%), millet (19.45%), and sesame (8.99%), while livestock occupying 4.26% of the area. This suggests a mixed crop‐livestock farming system potentially better aligned with subsistence and resilience goals. Conversely, some areas are characterized by narrow specialization. Vila Sede de Meconta with 66 540 ha allocates 24.80% of its area to sesame and 37.57% to soybean, with negligible use for other crops. Interestingly, livestock accounts for 6.07% of land use here, one of the highest shares among all sites.

Across all areas, crops like onions, tomatoes, cabbage, and spinach remain sparsely cultivated, typically accounting for less than 10% of the total area. This is likely due to their higher input and water demands. Rice, despite its dietary importance, is also marginal—occupying no more than 3%–4% of productive land—primarily due to the lack of irrigation infrastructure necessary for paddy cultivation. Other areas such as Teterranea show the highest proportion of land allocated to livestock (6.32%), despite minimal emphasis on specific crops, whereas Mpaco (2.17%), Murrutuma (0.17%), Nablusa (0.09%), Mutiva (0.01%), and Mutuzi (0.00%) negligible land use for livestock, underscoring sharp spatial disparities in animal production practices across the region.

**FIGURE 8:**
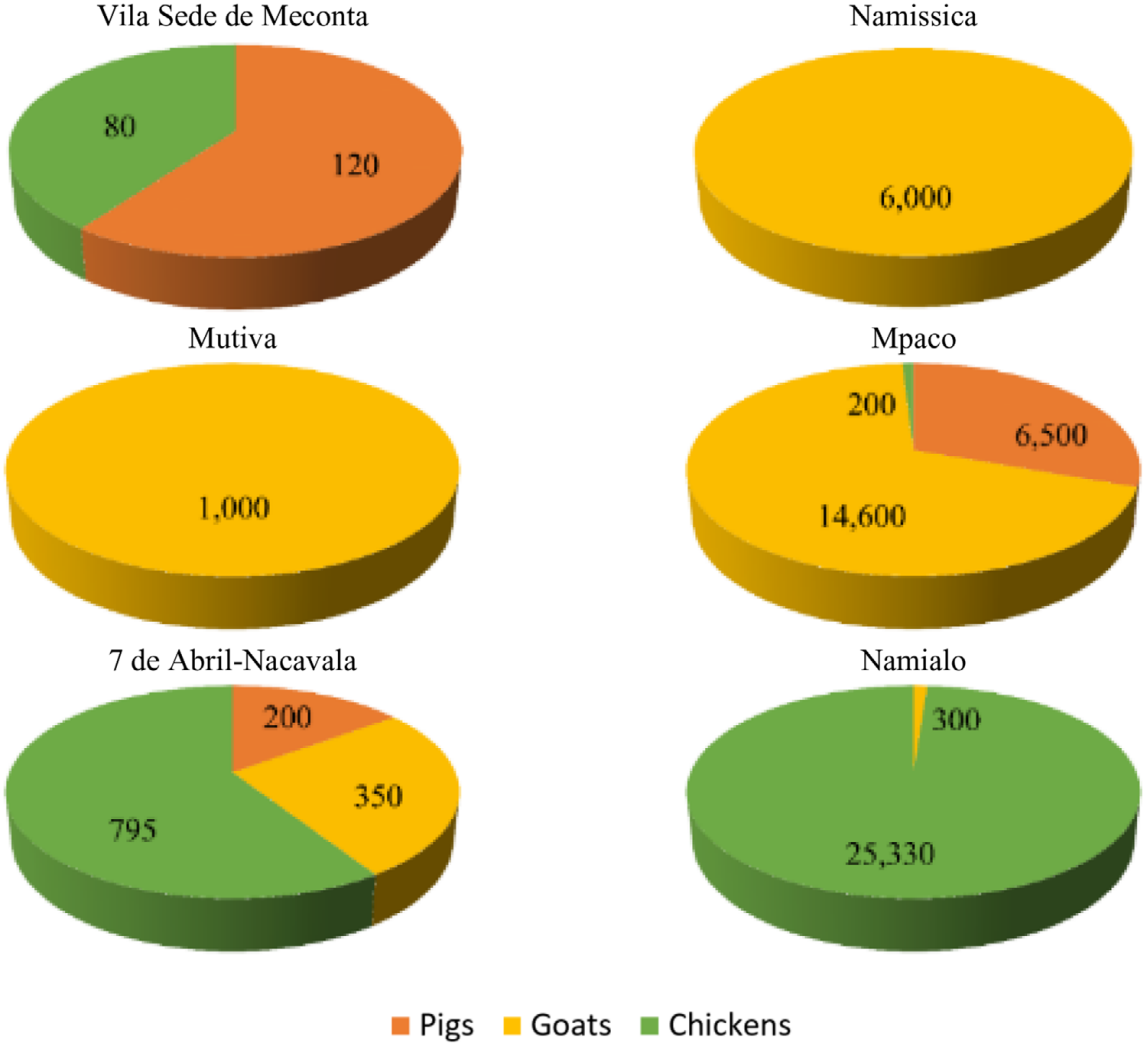
Livestock waste production across Meconta and Nacala districts. The data are reported as the kg of waste that is produced for each animal type (e.g., pigs, goats, chickens).

Concerning livestock waste production (Figure 8), Namialo records the highest output at 25 630 kg, with an overwhelming 99% derived from poultry and just 1% from goats. Mpaco follows closely with 21 300 kg, primarily composed of goat effluents (69%), while pigs contribute 31% and poultry only 1%. Namissica and Mutiva also report notable total livestock waste volumes—6000 and 1000 kg, respectively, both entirely attributable to goat farming, suggesting a reliance on small ruminants for animal production in these areas. In contrast, 7 de Abril‐Nacavala presents a lower total of 1345 kg, but with a more balanced distribution: 59% poultry, 26% goats, and 15% pigs, indicating a more diversified livestock system. Vila Sede de Meconta registers a modest 200 kg, with pigs accounting for (60%) and poultry (40%). No livestock waste production is recorded in Teterranea, Nablusa, Mutuzi, and Murrutumua, pointing to the absence of significant or intensive livestock activities in these localities. These findings underscore the heterogeneous nature of livestock waste generation across the region, shaped by differences in livestock type, production intensity, and management systems.

The distribution of livestock‐dedicated land across Meconta District reveals a highly uneven pattern of resource allocation. Vila Sede de Meconta (Meconta) and Teterranea (Nacala) together hold the largest individual shares, with 4040 ha (6.07% of the district's total livestock area) and 3700 ha (6.32%), respectively, establishing them as key hubs for animal production in the district. Namissica with 4500 ha, contributes the largest absolute area but represents a smaller proportion (4.26%) due to a larger total land base, indicating a more integrated crop‐livestock system. In contrast, Mpaco (2400 ha; 2.17%) and 7 de Abril‑Nacavala (3120 ha; 2.24%) play secondary roles, with moderate levels of livestock activity, while Namialo's 1601.5 ha (1.02%) and Murrutumua's 100 ha (0.17%) indicate only minimal livestock presence. At the lower end of the spectrum, Nablusa and Mutiva allocate just 50 ha (0.09%) and 6 ha (0.01%), respectively, while Mutuzi reports no land dedicated to livestock at all. This distribution highlights substantial spatial disparities in livestock development, likely influenced by factors such as land availability, water access, farming systems, and local production priorities.

### 3.3 GIS Analyses

#### 3.3.1 Territorial Distribution of Analyzed Data

**FIGURE 9:**
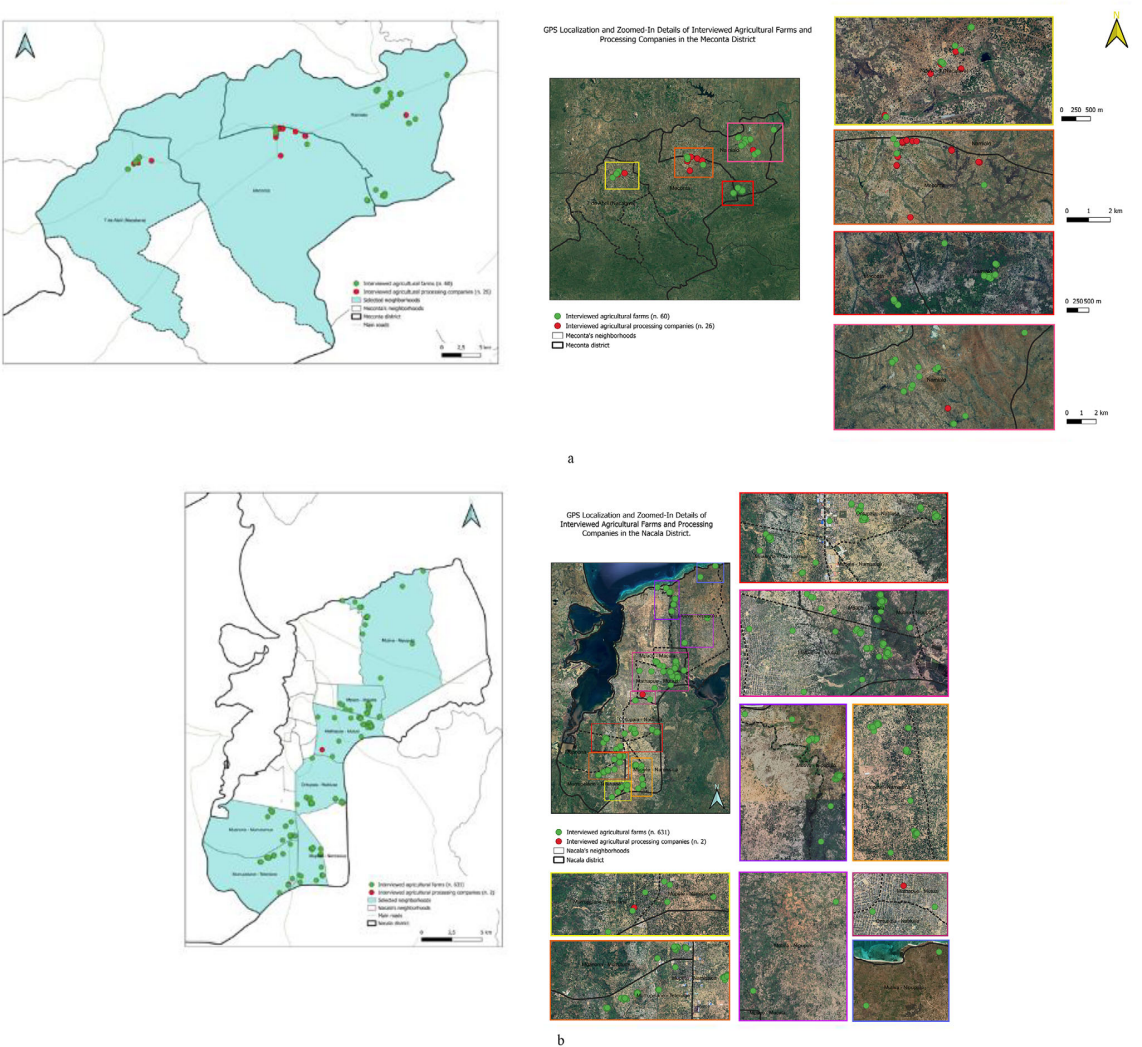
Spatial distribution of surveyed agricultural actors (farms and agro‐processing industries) in (a) Meconta and (b) Nacala districts.

Collected data were processed and standardized to create a unified geospatial database to enable comprehensive GIS‐based spatial analyses. All surveyed agricultural entities—including farms and agro‐processing industries—were georeferenced using GPS coordinates (Figure 9), providing a precise spatial foundation. This geospatial layer was further enriched with primary survey responses and composite indicators derived from the cleaned dataset, facilitating a detailed, multidimensional territorial analysis of agricultural and livestock patterns in the Meconta and Nacala districts. To preserve geographic specificity, all assessments were conducted separately for each district.

Figure 9 illustrates the contrasting spatial distribution of agricultural actors within the two districts. In Meconta (Figure 9a), 60 farms and 26 agro‐processing industries cluster prominently along primary transportation routes and within key urban centers such as Namialo and 7 de Abril – Nacalava. This spatial concentration suggests a synergistic relationship between agricultural production and processing nodes, most probably supported by accessible transport infrastructure and fostering a more integrated agri‐food system.

In stark contrast, Nacala (Figure 9b) exhibits a markedly different spatial and structural pattern. Although the district hosts a substantially higher number of farms (631), it contains only two agro‐processing industries. The farms are more geographically dispersed but still align along major road networks and are concentrated in neighborhoods like Murrutumua, Mpaco, and Mutiva. This pronounced imbalance highlights a critical bottleneck in the agricultural value chain, where robust primary production is undermined by an underdeveloped local processing infrastructure, potentially forcing producers to depend on external markets for downstream activities.

These divergent spatial dynamics reflect fundamentally different agro‐economic structures. Meconta benefits from a more balanced and interconnected agro‐industrial ecosystem with tighter linkages between farming and processing, while Nacala's landscape is characterized by a strong farming base but limited local industrial transformation capacity. This contrast underscores distinct development trajectories with significant implications for regional agricultural policy and investment priorities.

**FIGURE 10:**
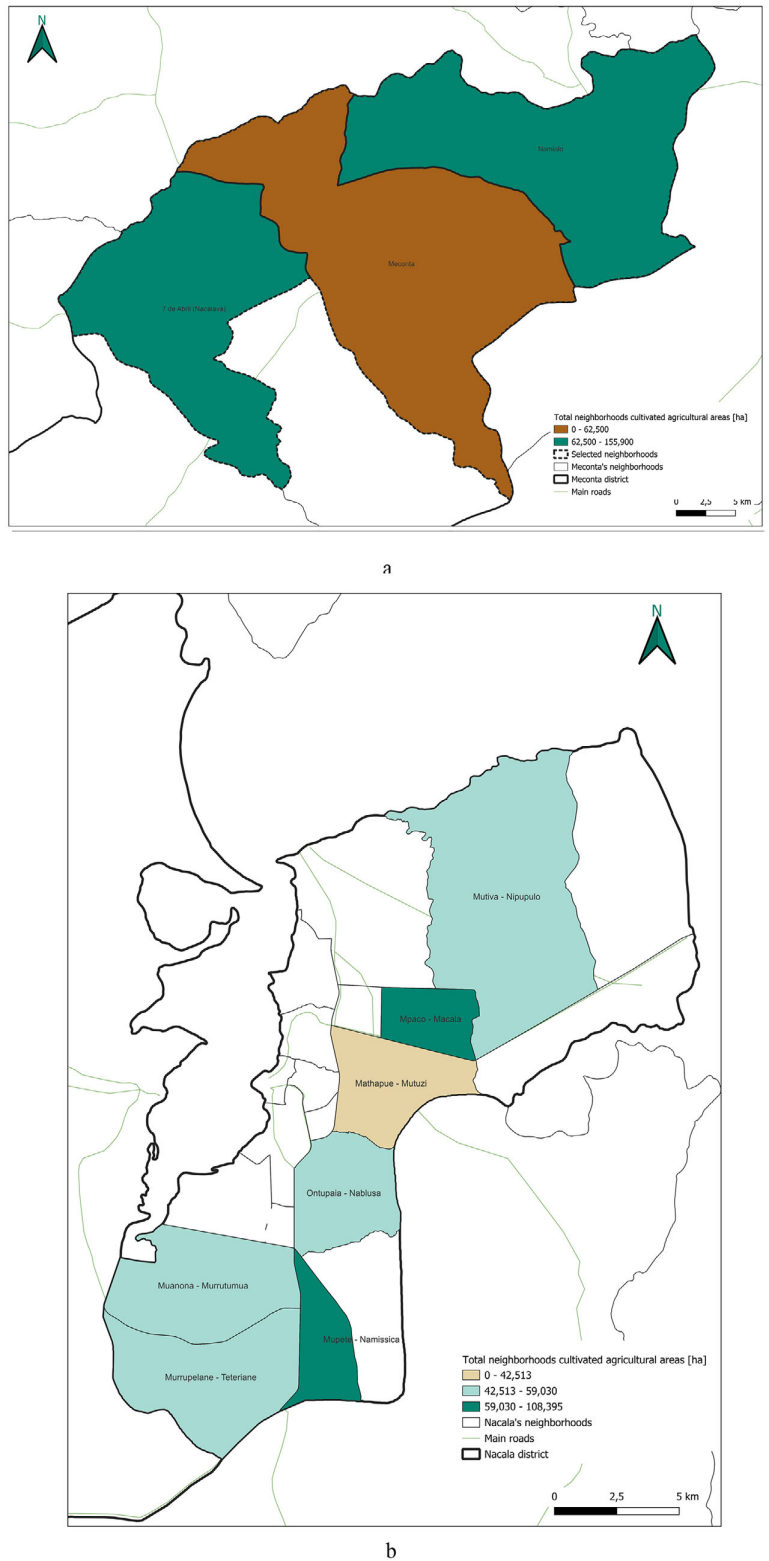
Distribution of cultivated agricultural areas in (a) Meconta and (b) Nacala districts.

The spatial analysis of cultivated agricultural land (Figure 10) highlights important territorial distinctions in agricultural intensity between Meconta and Nacala districts.

In Meconta (Figure 10a), agricultural land use is concentrated in peripheral neighborhoods, particularly Namialo and 7 de Abril‐Nacalava, which fall into the upper intensity class (62 500–155 900 ha). The central Meconta neighborhood, by contrast, remains in the lower land‐use class (0–62 500 ha), suggesting limited cultivation activity. This bimodal pattern indicates that only a few areas drive the district's agricultural performance, probably linked to more favorable agroecological conditions or better land access at the margins of the district.

In Nacala (Figure 10b), a more diversified and evenly distributed land‐use pattern emerges, with three cultivation classes (0–42 513, 42 513–59 030, and 59 030–108 395 ha). Key neighborhoods such as Mpaco and Namissica are clearly the most agriculturally active zones, corresponding with other high‐performing indicators like production output and residue generation. The overall fragmentation and spatial spread of cultivated areas in Nacala may reflect a smallholder‐dominated agricultural system and heterogeneous land tenure structures, in contrast to Meconta's more consolidated but spatially limited cultivation footprint.

**FIGURE 11:**
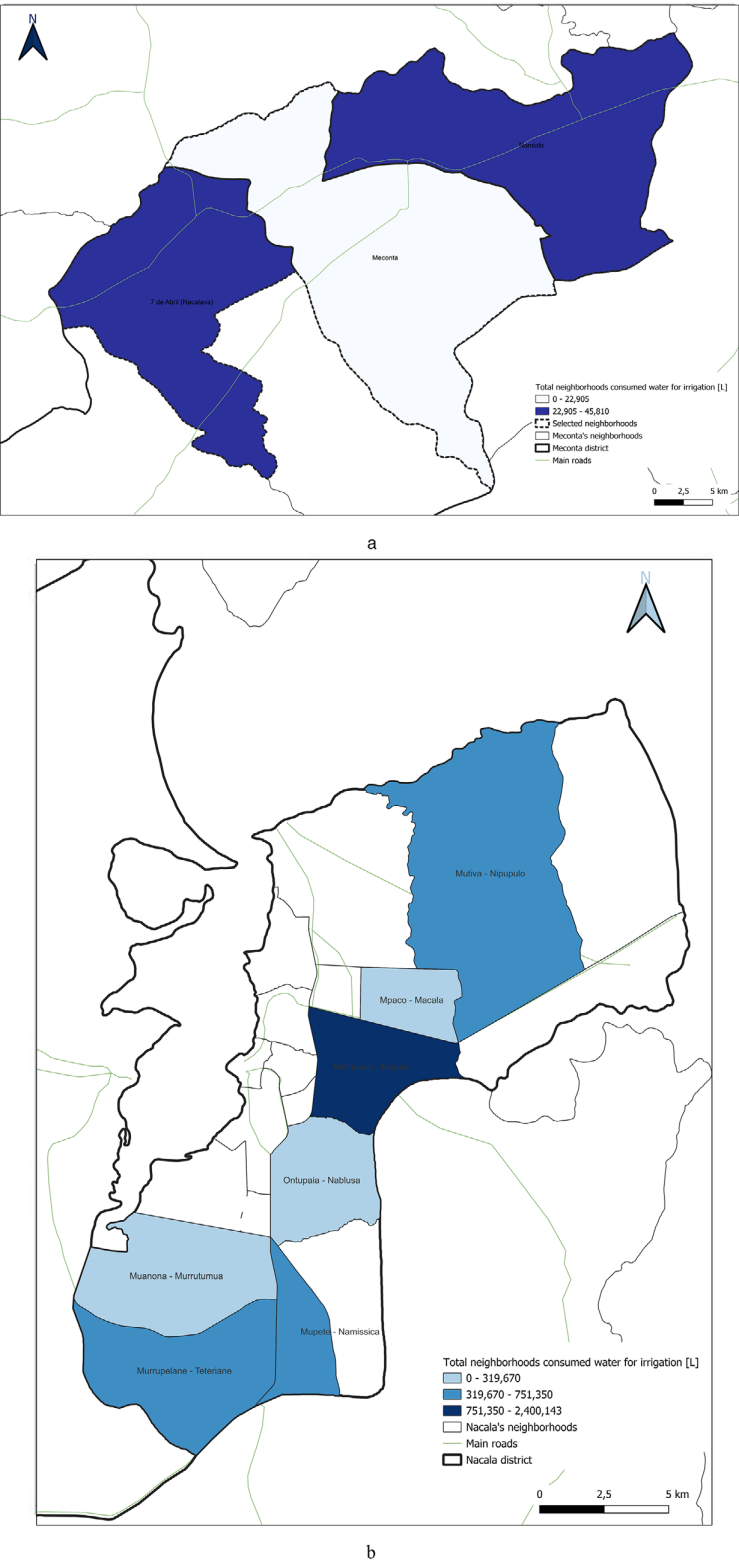
Irrigation water consumption across neighborhoods in (a) Meconta and (b) Nacala districts.

**FIGURE 12:**
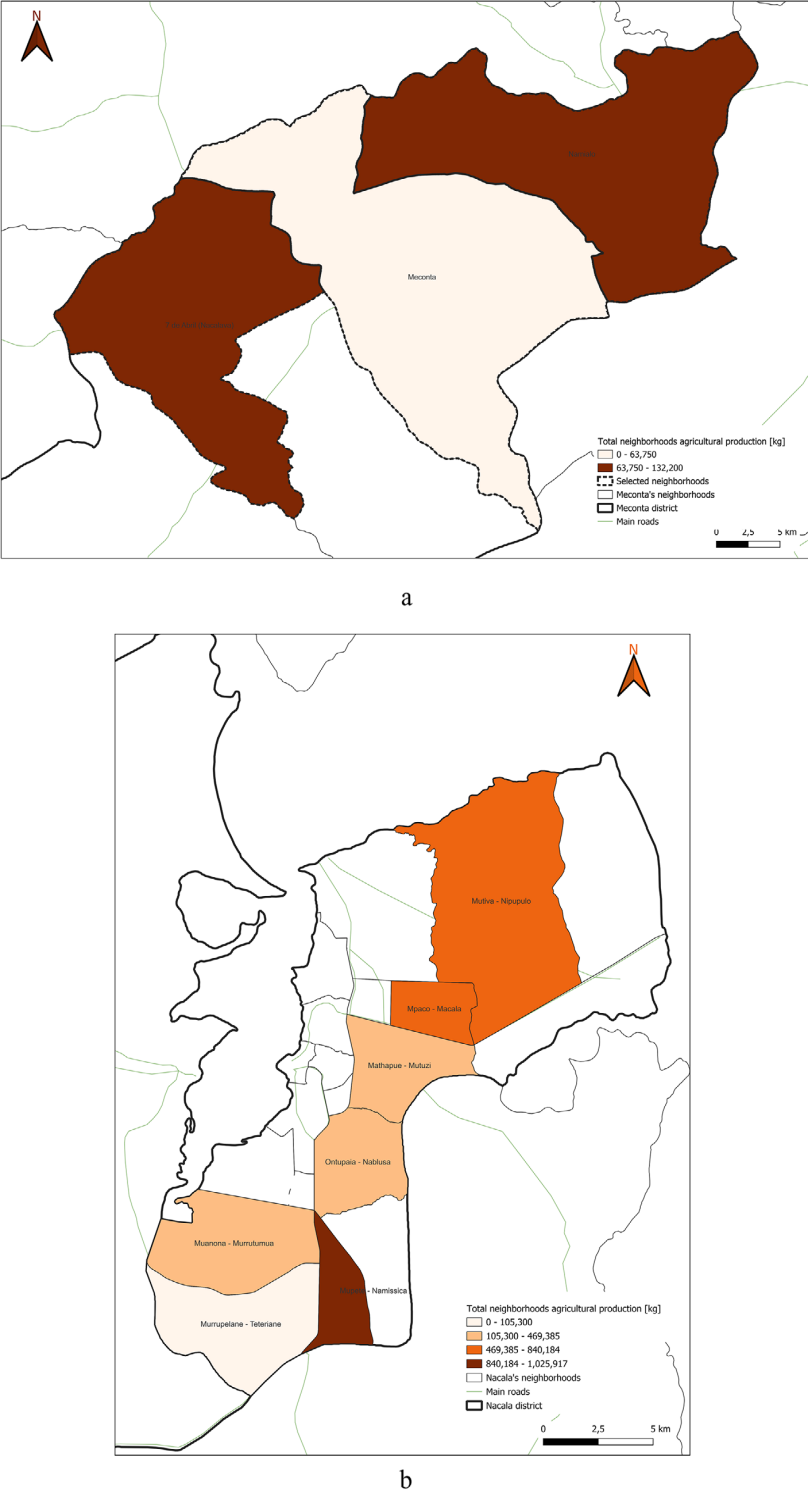
Total agricultural production per neighborhood in (a) Meconta and (b) Nacala districts.

**FIGURE 13:**
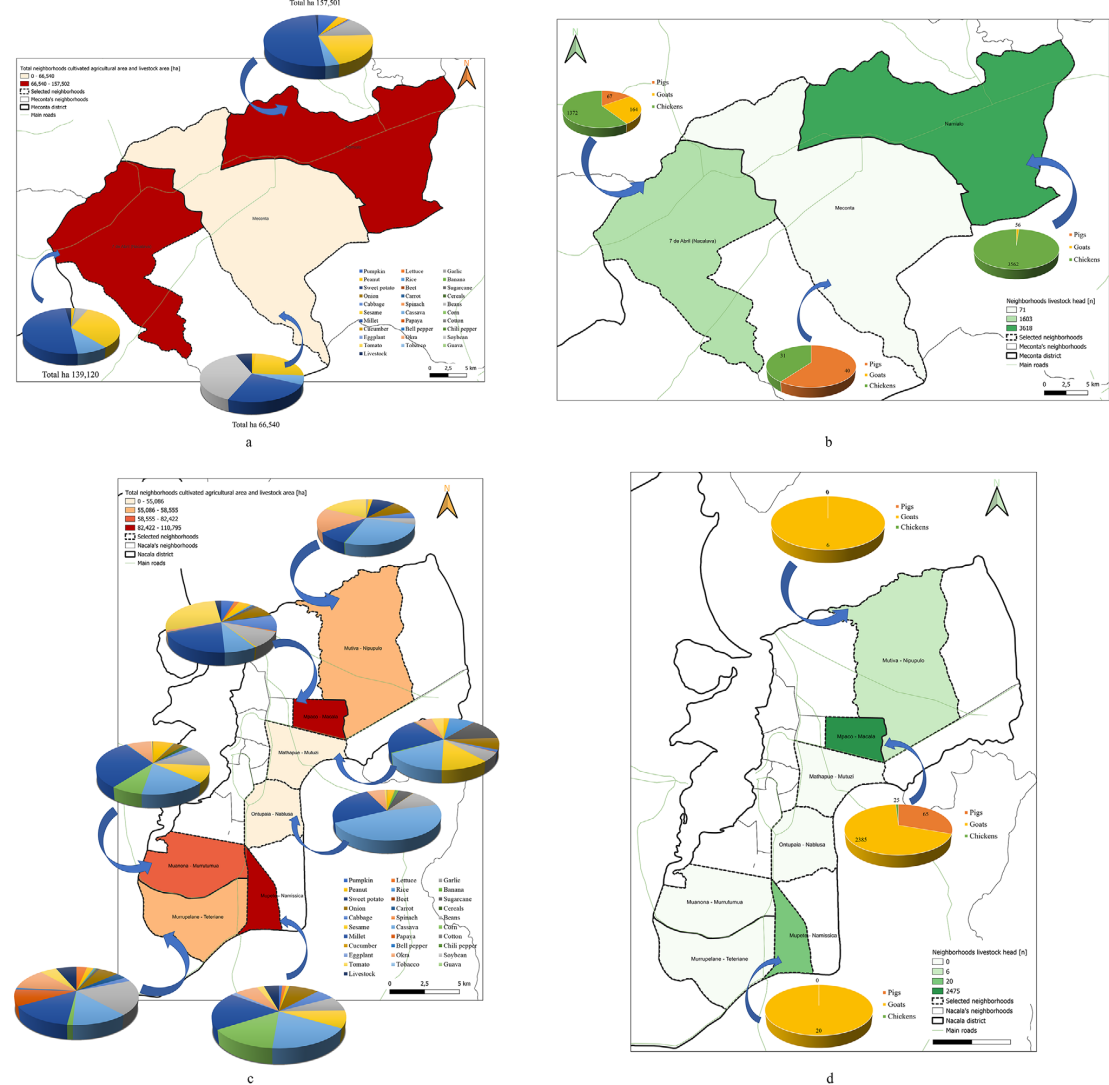
Spatial distribution of (a, c) livestock‐related land use and (b, d) livestock headcount in Meconta and Nacala districts.

**FIGURE 14:**
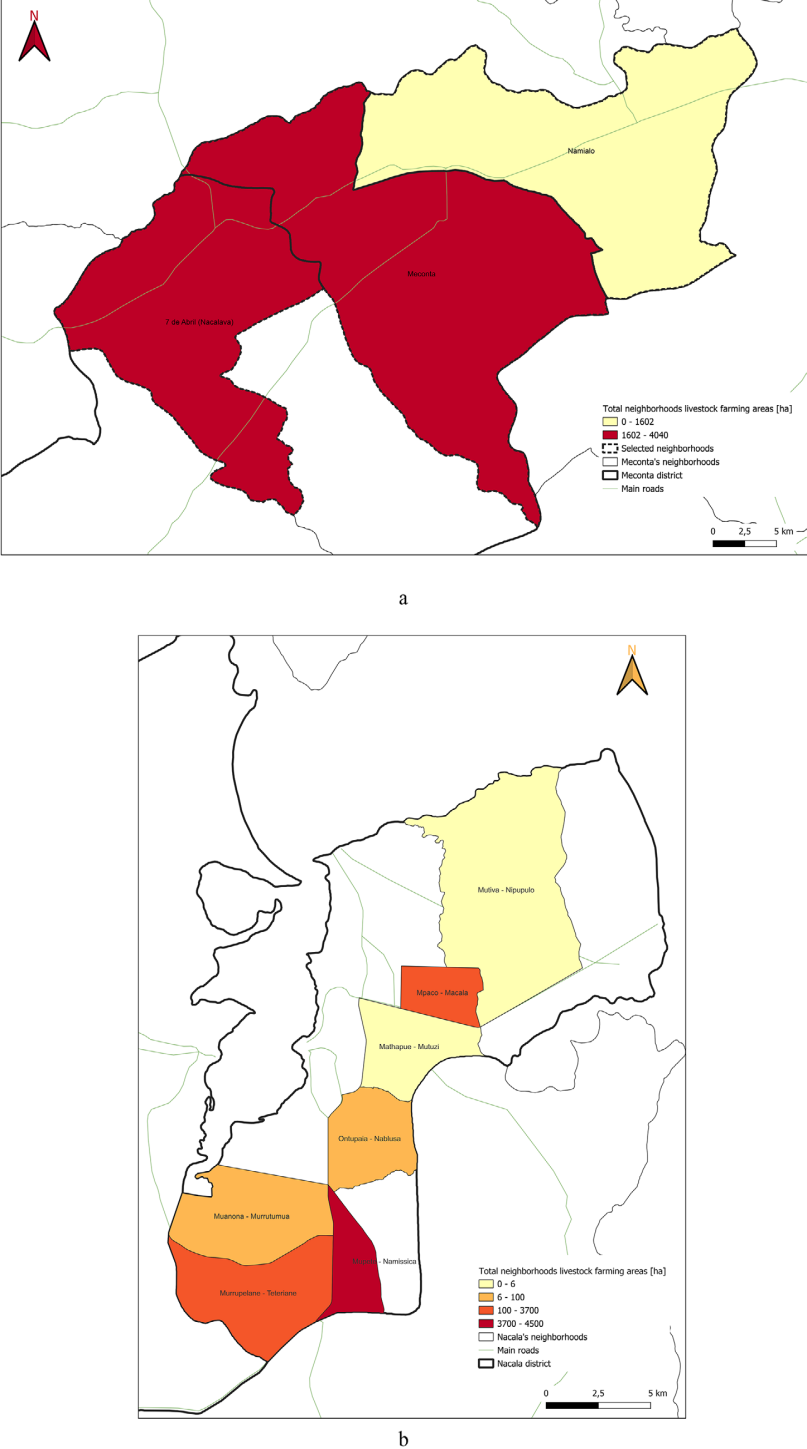
Total land dedicated to livestock farming in (a) Meconta and (b) Nacala districts.

**FIGURE 15:**
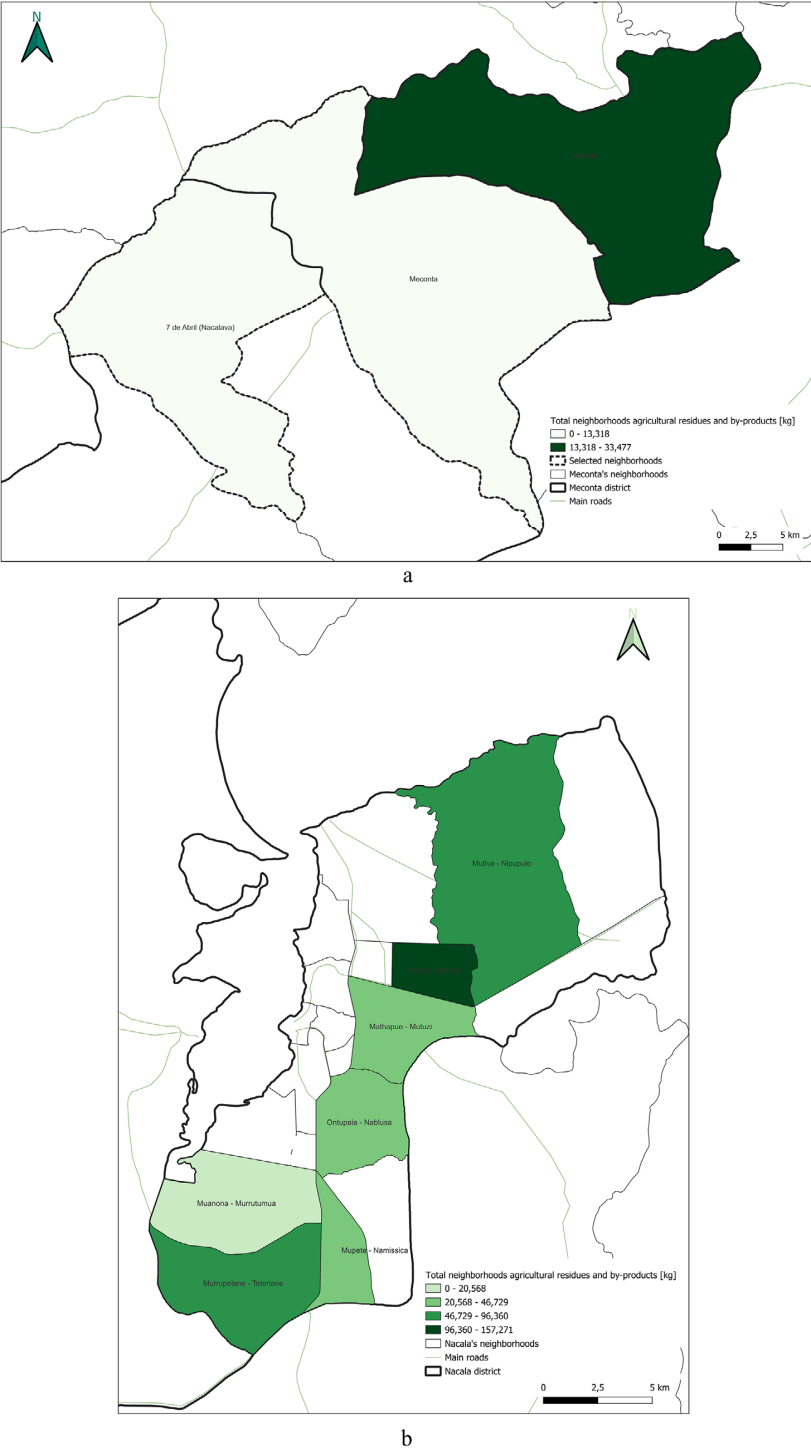
Agricultural residues and by‐product availability in (a) Meconta and (b) Nacala district.

**FIGURE 16:**
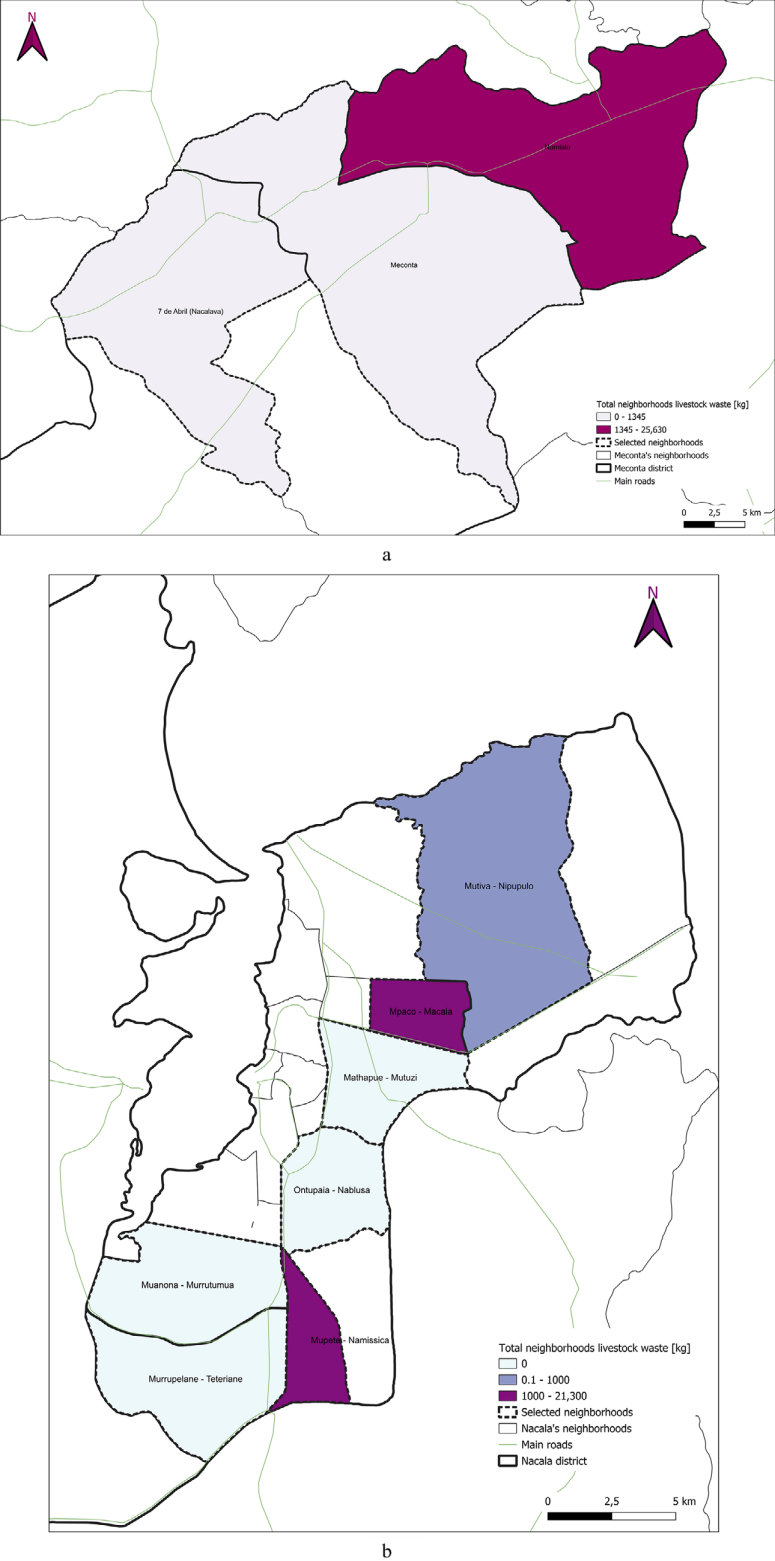
Livestock waste availability per neighborhood in (a) Meconta and (b) Nacala districts.

This spatial land‐use distribution aligns closely with patterns observed in irrigation water consumption (Figure 11), production output (Figure 12), livestock‐related land use and farming (Figures 13 and 14), as well as residue and livestock waste availability (Figures 15 and 16). In Meconta, production is tightly linked to a few cultivated hotspots, particularly Namialo and 7 de Abril‐Nacalava. In Nacala, the more dispersed but intensive cultivation suggests a broader agricultural base and potentially more resilience to shocks or localized resource constraints.

In summary, while Meconta's agriculture appears spatially concentrated and possibly constrained by access or infrastructure, Nacala benefits from diverse, distributed cultivation, likely supported by better irrigation, crop diversity, and higher input usage, but also requiring greater coordination for sustainable land management.

Another critical parameter analyzed at the territorial level—based on data from stakeholder interviews—was irrigation water consumption, which provides insights into agricultural intensity, crop water demand, and resource accessibility (Figure 11). The comparison between Meconta and Nacala districts reveals stark differences in both the volume and spatial distribution of irrigation use.

In Meconta, irrigation remains modest and spatially limited, with only the neighborhoods of Namialo and 7 de Abril‐Nacalava reaching the upper range of consumption (22 905–45 810 L) (Figure 11a). The central neighborhood of Meconta records even lower usage (<22 905 L), indicating a predominantly rainfed agricultural system or the cultivation of low‐water‐demand crops such as millet and sesame. This limited irrigation footprint may also reflect constraints in infrastructure or water resource availability, further contributing to the district's overall lower agricultural productivity.

In sharp contrast, Nacala exhibits substantially higher irrigation volumes, pointing to more intensive or extensive cultivation practices. The neighborhood of Mutuzi stands out with peak usage exceeding 2.4 million liters, the highest recorded in the analysis (Figure 11b). Other areas such as Teterranea, Mutiva, and Mpaco also demonstrate significant water consumption (319 670–751 350 L), consistent with the district's high‐output zones and crop diversification. This pattern suggests either the cultivation of water‐intensive crops (e.g., sugarcane, onions, cabbage) or better access to surface or groundwater resources and irrigation technologies.

The stark contrast in irrigation patterns between the two districts reflects their agricultural profiles: while Meconta relies largely on low‐input, rainfed systems with minimal irrigation infrastructure, Nacala supports more productive, irrigated farming systems, likely enabled by greater water availability, better agronomic planning, and possibly external support or investment. However, the intensive water use in Nacala also raises concerns about resource sustainability, especially if water management practices are not carefully monitored.

Building upon the assessment of cultivated areas, an evaluation of agricultural production levels and resulting by‐products was conducted to understand spatial disparities and valorization potential (Figure 12). The analysis reveals marked contrasts between the Meconta and Nacala districts, not only in the volume of production, but, also, in its distribution and concentration. In Meconta, most neighborhoods fall into the lowest production category (0–63 750 kg), reflecting generally low agricultural yields. Only 7 de Abril‐Nacavala and Namialo, both peripheral neighborhoods, exceed this threshold, underscoring the limited productivity of the district (Figure 12a). This low output aligns with earlier findings on land use and crop specialization, where millet and sesame dominate but do not translate into high‐volume production. The relatively limited integration of livestock and the modest use of high‐value crops, such as soybeans (confined to Vila Sede de Meconta), further constrain overall agricultural output.

By contrast, Nacala shows a broader range and higher intensity of production, with several neighborhoods far surpassing Meconta's maximum levels (Figure 12b). The Namissica area reaches the highest yield class (840 184–1 025 917 kg), driven by both the extent of cultivation and crop diversity. Mpaco and Mutiva also register significant yields (469 385–840 184 kg), suggesting more intensive, better‐resourced, or technically supported farming systems. This pattern aligns with land use trends that highlight Nacala's diversified and locally intensive agriculture, particularly in neighborhoods with large cultivated areas and moderate to high levels of crop diversity.

These disparities in production strongly correlate with the potential quantity and type of agricultural by‐products available for valorization. In Nacala, high‐output zones offer greater volumes of organic residues (e.g., cassava peels, corn husks, sugarcane bagasse), presenting viable opportunities for composting, bioenergy, or feed production. In Meconta, the limited production restricts the generation of such by‐products to a few hotspots like Namialo and 7 de Abril‐Nacalava, indicating the need for localized or small‐scale valorization strategies.

Overall, the comparative analysis confirms that Nacala benefits from more favorable agro‐ecological and infrastructural conditions, enabling higher productivity and by‐product generation. Meanwhile, Meconta's consistently low yields reflect systemic constraints that may include limited access to inputs, lower soil fertility, or weaker extension services. These findings underscore the need for targeted interventions in Meconta to enhance productivity and promote sustainable by‐product utilization.

An integrated assessment of cultivated land and livestock‐related areas in the Meconta and Nacala districts reveals pronounced spatial and functional differences in agricultural practices, crop specializations, and livestock systems (Figures 13 and 14).

In Nacala, the largest cultivated areas are found in Mpaco (110 975 ha) and Namissica (106 713 ha), where millet, cassava, and corn dominate, reflecting their adaptation to local conditions. Mpaco shows notable agricultural diversification, with crops such as beans, onion, sweet potato, cabbage, and sugarcane. Teterranea (58 555 ha) also stands out for its significant livestock allocation, indicating a mixed farming system. Other areas, like Mutiva (56 201 ha), Murutumua (59 080 ha), and Nablusa (53 970 ha), predominantly grow millet and cassava, while Mutuzi (42 513 ha) specializes in sugarcane, beet, and cassava, suggesting more intensive practices. In Meconta, 7 de Abril‐Nacavala has the largest cultivated area (199 120 ha), followed by Namialo (157 501.5 ha) and Vila Sede de Meconta (66 540 ha). Millet is the dominant crop, especially in Namialo, where it covers over 50% of land. Sesame is also prominent (35% in 7 de Abril‐Nacavala, 25% in Namialo). Vila Sede de Meconta is unique for its 35% soybean cultivation, nearly absent elsewhere. Beans and pumpkins are important in Namialo, where beans alone occupy around 20% of land. Cassava appears throughout the district but is most prevalent in 7 de Abril‐Nacavala.

The inclusion of livestock land adds another dimension to the land‐use profile (Figures 13a,c and 14a,b). In Meconta, livestock land use is more uniformly integrated across neighborhoods, though actual land shares remain modest. The central Meconta neighborhood allocates 12% of its 66 540 ha to livestock—the highest in the district—despite specializing in soybean and sesame. In contrast, Namialo allocates only 2% of its land to livestock and 7 de Abril‐Nacavala about 5%.

In Nacala, livestock land use is more fragmented and sparser. Only Teterranea (6%) and Namissica (4%) show notable allocations, while key agricultural hubs like Mpaco and Mutiva report 0% livestock land use. Despite Namissica's crop diversity, livestock remains marginal. Likewise, Mutuzi and Nablusa, each with over 40 000 ha of productive land, also report no livestock use.

Figure 14 further illustrates these disparities: Meconta neighborhoods such as Meconta and 7 de Abril fall into the highest livestock land use category (1602–4040 ha), while Namialo remains in the lowest. In Nacala, only Namissica exceeds 3700 ha, while most neighborhoods are either moderate (100–3700 ha) or minimal (<100 ha). Livestock headcount data (Figure 13b,d) reveal that land allocation does not always align with actual animal populations, indicating potential underutilization or informal practices.

In Meconta, Namialo hosts the highest number of chickens (3562), despite only 2% land allocation, implying intensive backyard poultry. 7 de Abril‐Nacavala supports a mix of livestock: 67 pigs, 164 goats, and 1372 chickens. In contrast, the Meconta neighborhood, while having the most land dedicated to livestock (12%), reports only 40 pigs and 31 chickens, suggesting low livestock density or underused grazing capacity.

In Nacala, livestock numbers are generally low but sometimes incongruent with land use data. Mpaco, for example, has zero livestock land allocation yet hosts the highest goat population (2385 goats), alongside 65 pigs and 25 chickens. This points to informal or nomadic grazing systems. Other areas, like Mutiva (6 goats) and Namissica (20 goats), show minimal livestock presence, confirming limited integration.

Overall, Meconta demonstrates a more systematic and integrated approach to livestock farming, with relatively higher and more evenly distributed land allocations and headcounts. However, mismatches between land use and livestock numbers suggest low‐density or underutilized systems. Conversely, Nacala's agricultural system is crop‐dominant, with livestock activity being patchy and localized, often occurring without corresponding land allocation. These patterns underscore the need for differentiated planning strategies: one focused on enhancing livestock efficiency in Meconta, and another on formalizing and supporting informal livestock systems in Nacala.

The spatial analysis of agricultural residues and livestock waste (Figures 15 and 16) reveals significant differences between Meconta and Nacala districts, reflecting broader disparities in cropping intensity, livestock distribution, and agro‐ecological systems. Based on cultivated area, production volumes, and livestock distribution, both agricultural residues and by‐products, as well as livestock waste, show distinct territorial patterns.

In Meconta (Figure 15a), agricultural residues are highly localized, with Namialo emerging as the only area with notable residue volumes (13 318–33 477 kg). The remainder of the district, including 7 de Abril‐Nacalava and central Meconta, registers significantly lower outputs (0–13 318 kg), suggesting limited crop diversity or lower production intensities across most of the territory. Conversely, Nacala presents a more spatially diverse and quantitatively higher residue profile (Figure 15b). The highest concentrations are found in Mpaco and Mutiva, with volumes reaching up to 157 271 kg. Several other neighborhoods—such as Teterranea and Mutuzi—also report moderate levels of residue generation, pointing to a more intensive and heterogeneous agricultural landscape. These patterns underscore Nacala's broader crop base and higher productivity, which also suggest greater potential for residue valorization and circular economy applications.

Livestock waste generation follows a similar district‐level divergence (Figure 16). In Meconta (Figure 16a), only Namialo again stands out, with waste volumes ranging from 1345 to 25 630 kg, while all other neighborhoods produce minimal quantities (0–1345 kg). In contrast, Nacala (Figure 16b) demonstrates a more dispersed pattern of livestock waste generation. Although most areas report low outputs (0.1–1000 kg), three localities—Mpaco, Namissica, and Mutiva—exhibit significantly higher volumes, reaching up to 21 300 kg. This more even spatial distribution may reflect better‐integrated or more diversified animal husbandry systems, and potentially more favorable conditions for decentralized waste‐to‐resource initiatives such as biogas production or composting.

Together, Figures 15 and 16 indicate that while Meconta's resource recovery potential is largely concentrated in Namialo, Nacala offers multiple hotspots for agricultural residue and waste utilization, suggesting a greater scope for implementing circular economy strategies at a district‐wide scale.

**FIGURE 17:**
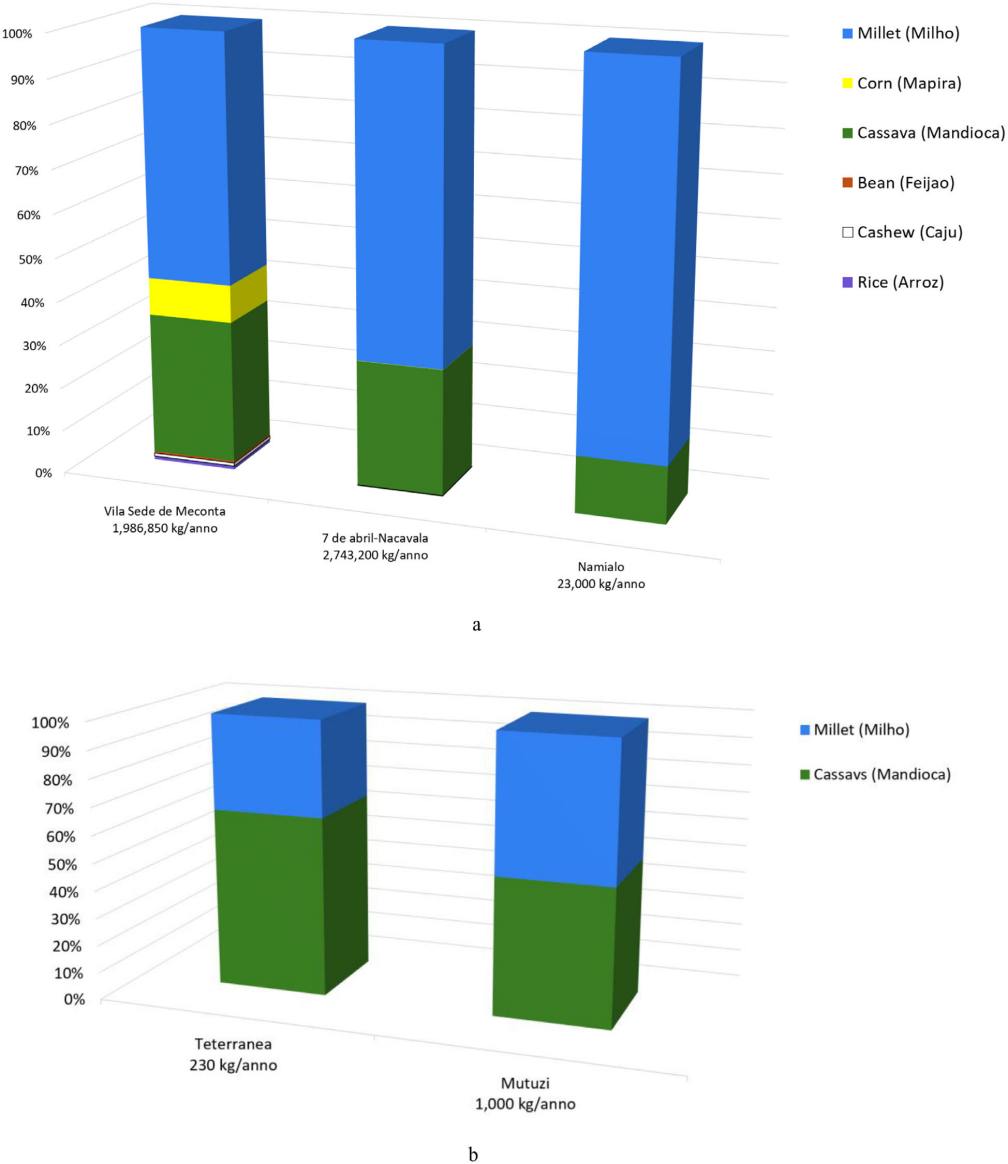
Annual volume and crop type of processed products in (a) Meconta and (b) Nacala districts.

Finally, the analysis of processed product volumes and crop prioritization (Figure 17) confirms a significant disparity between the agro‐industrial capacities of Meconta and Nacala districts. In Meconta (Figure 17a), processing volumes are markedly higher, particularly in the neighborhoods of 7 de Abril‐Nacavala and Vila Sede de Meconta, where annual outputs reach approximately 2 743 200 and 1 986 850 kg, respectively. Namialo, though smaller in scale, still contributes about 23 000 kg annually.

The processing landscape in Meconta is dominated by millet, which constitutes over 50% of total processed products in 7 de Abril‐Nacalava and Vila Sede, and exceeds 70% in Namialo. Cassava follows as the second most significant crop across all three locations. Notably, Vila Sede de Meconta also processes a modest range of additional crops—corn (around 10% of its total volume), along with beans, cashew, and rice (each under 5%)—indicating a relatively diversified agro‐processing sector and a broader capacity for value addition.

In contrast, Nacala's agro‐industrial development remains rudimentary (Figure 17b). Only two neighborhoods—Mutuzi and Teteranea—reported any processing activity, with annual volumes limited to 1000 and 230 kg, respectively. Processing in both areas is confined to just two crops: cassava and millet. In Mutuzi, cassava accounts for 60% (600 kg/year) and millet 40% (400 kg/year) of total processed volume. In Teteranea, cassava dominates even more strongly, representing 70% (161 kg/year) compared to 30% (69 kg/year) for millet. The narrow product range and low output volumes underscore the limited industrial infrastructure in Nacala, despite its high levels of primary agricultural production. This disparity highlights an unrealized potential for agro‐industrial expansion and suggests a strategic opportunity to enhance local value chains and reduce post‐harvest losses through improved processing capabilities.

The spatial analysis reveals two districts with divergent agricultural and livestock development trajectories. Meconta showcases a more integrated and compact system, with higher levels of agro‐processing, moderate livestock activity, and spatially concentrated agricultural output. Its structure points to opportunities for intensification and value‐chain consolidation. In contrast, Nacala demonstrates a broad and dynamic agricultural base characterized by high production, greater water usage, and dispersed residue generation. However, the lack of agro‐processing and limited livestock integration suggests structural weaknesses in downstream capacity and diversification. This indicates an urgent need for investment in post‐harvest infrastructure, value‐added processing, and livestock systems. Together, the spatialized data build a compelling narrative of regional contrasts, informing targeted development strategies and investment priorities tailored to the unique geographies of Meconta and Nacala.

#### 3.3.2 Potentials and Challenges Areas: Heatmaps

In this section, data were processed using the spatial analytical tools available in QGIS to generate tailored heatmaps. These visual outputs aim to highlight both areas of opportunity and areas facing critical challenges for the deployment of innovative water–energy technologies. The primary objective of this GIS‐based approach was to spatially identify zones where such technologies could have the greatest impact—either due to enabling conditions (e.g., resource availability, population density) or urgent needs (e.g., water scarcity, energy deficiency).

By transforming complex datasets into intuitive spatial visualizations, heatmaps serve as powerful decision‐support tools for stakeholders such as policymakers, engineers, planners, and investors. Heatmaps include cultivated surface area, agricultural production volume, irrigation water consumption, and the quantity of available agricultural waste and by‐products.

This methodology builds on prior research that emphasizes the integration of spatial analysis and participatory approaches in resource management [6, 13, 14], extending these concepts to the context of rural Mozambique. While previous studies have highlighted the potential of biochar and waste valorization for environmental remediation and climate mitigation, this study contributes by linking residue availability directly with water‐energy dynamics in a geospatially explicit framework, providing actionable insights for planning sustainable interventions.

**FIGURE 18:**
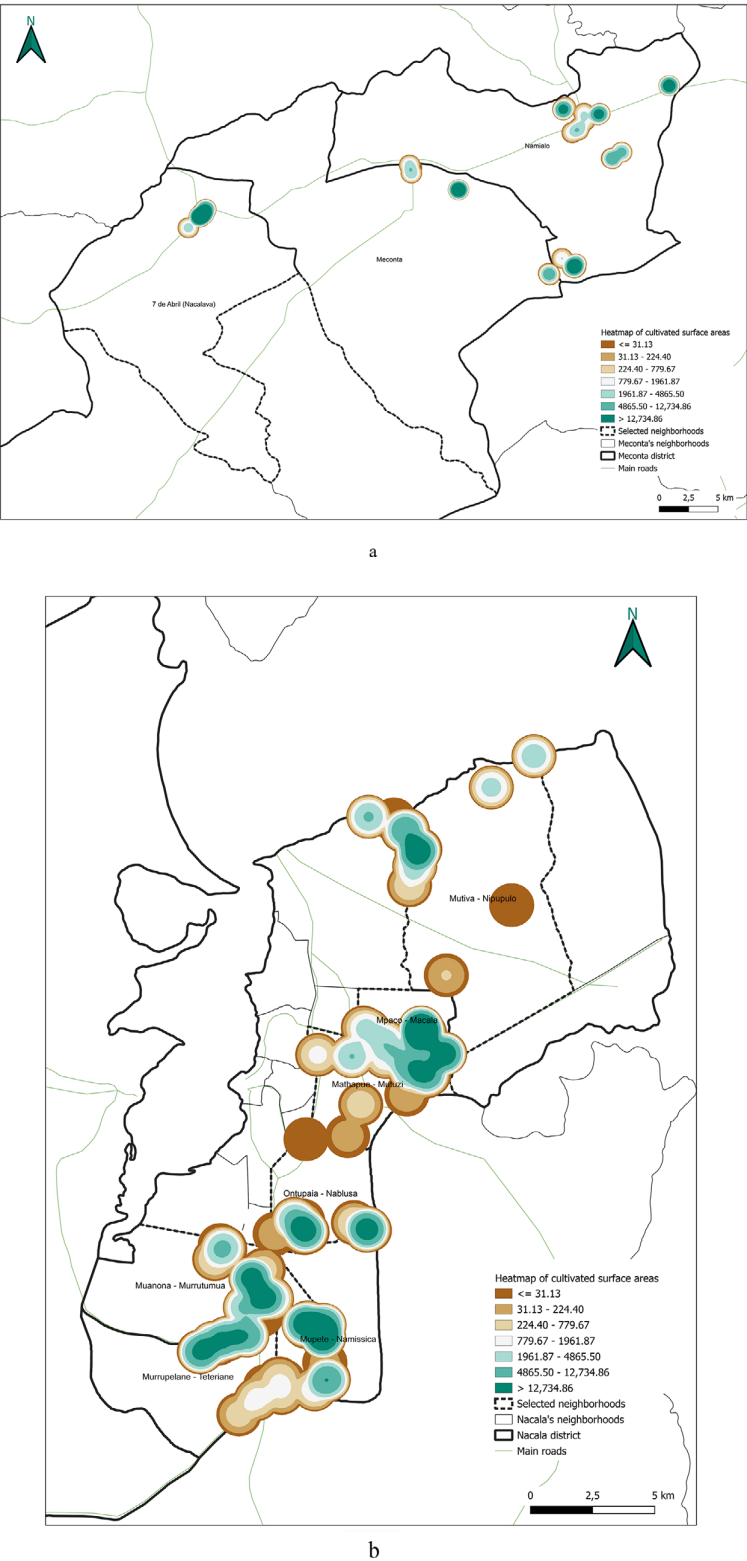
Heatmap of cultivated surface areas in Meconta (a) and Nacala (b) districts.

The heatmaps for cultivated surface area in Nacala and Meconta reveal distinct regional patterns of agricultural land use intensity (Figure 18).

In Meconta (Figure 18a), the north‐eastern and south‐eastern regions, particularly near Namialo, exhibit the most extensive cultivated areas. Smaller yet intensive agricultural clusters are also observed in the northwest. Conversely, the southwestern part of the district, including 7 de Abril‐Nacalava, remains largely underutilized, with cultivated land falling in the lowest class (≤31.13 ha). These spatial disparities suggest uneven land development and possible inefficiencies in resource allocation.

In Nacala (Figure 18b), agricultural activity is highly concentrated in the southern and central neighborhoods—such as Murrutumua, Murrupelane, Teterranea, and Namissica—where cultivated areas often exceed 12 734.86 ha, falling into the highest intensity class. In contrast, the northern neighborhoods such as Mutiva show minimal cultivation, pointing to underutilized land and potential development opportunities.

**FIGURE 19:**
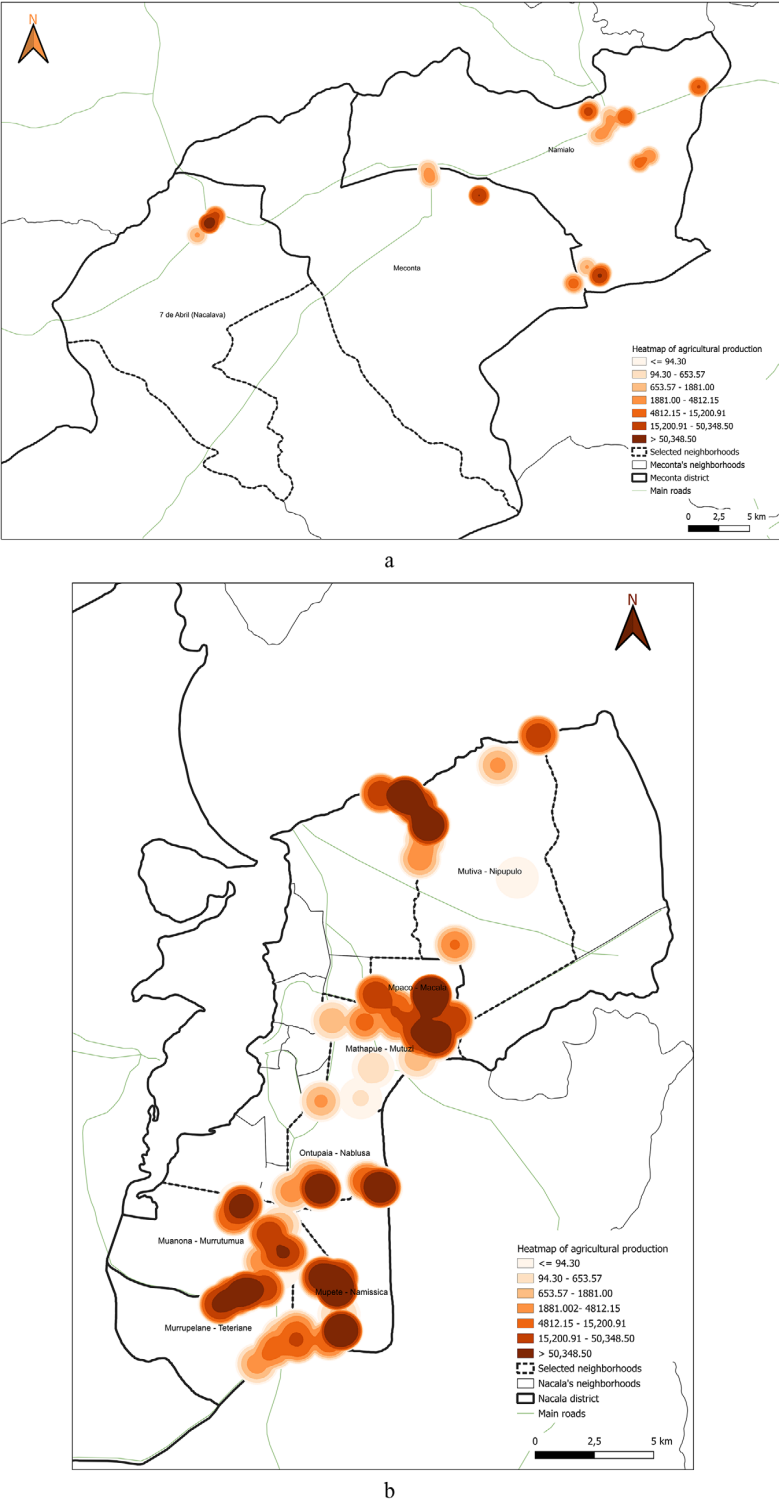
Heatmap of agricultural production volumes in Meconta (a) and Nacala (b) districts.

The heatmaps depicting agricultural production volumes further illustrate spatial heterogeneity across the two districts (Figure 19).

In Meconta (Figure 19a), the highest production levels—exceeding 15 200.91 tons—are concentrated in the north‐eastern and south‐eastern areas near Namialo. Meanwhile, vast portions of the district, including 7 de Abril‐Nacalava, fall within the lowest class (≤94.30 tons), indicating low productivity despite available land.

In Nacala (Figure 19b), the production distribution is broader and more consistent, with the southern and central neighborhoods such as Namissica and Teterranea, showing high outputs (above 50 348.50 tons). These regions represent intense agricultural activity, supported by both extensive cultivation and infrastructure.

**FIGURE 20:**
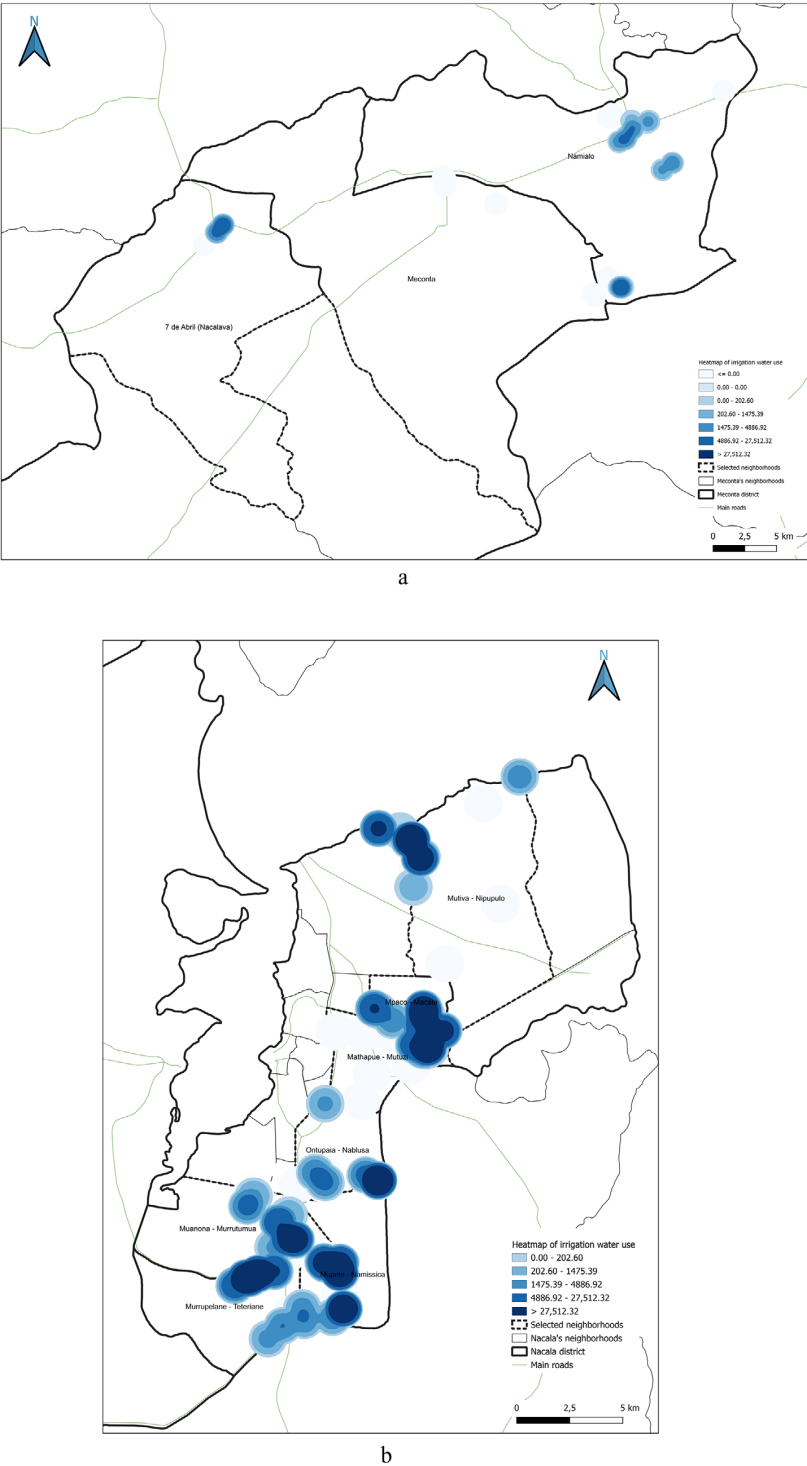
Heatmap of irrigation water consumption in Meconta (a) and Nacala (b) districts.

The comparison of irrigation water consumption (Figure 20) highlights substantial differences in water use patterns between the two districts, helping to identify areas of concern.

In Meconta (Figure 20a), irrigation levels are generally low. Even in Namialo, which has the largest cultivated area (157 501 ha), water use remains moderate, probably due to the prevalence of drought‐resistant crops such as millet and sesame. Neighborhoods such as Meconta town and 7 de Abril‐Nacalava consume less than 1500 m^3^, suggesting limited irrigation infrastructure or reliance on rain‐fed agriculture.

By contrast, Nacala (Figure 20b) demonstrates more intensive irrigation use. Neighborhoods such as Mpaco and Namissica exceed 27 500 m^3^ in water consumption, probably reflecting the cultivation of water‐intensive or higher‐value crops. This implies a more diversified and resource‐intensive agricultural system, possibly benefiting from improved water access.

**FIGURE 21:**
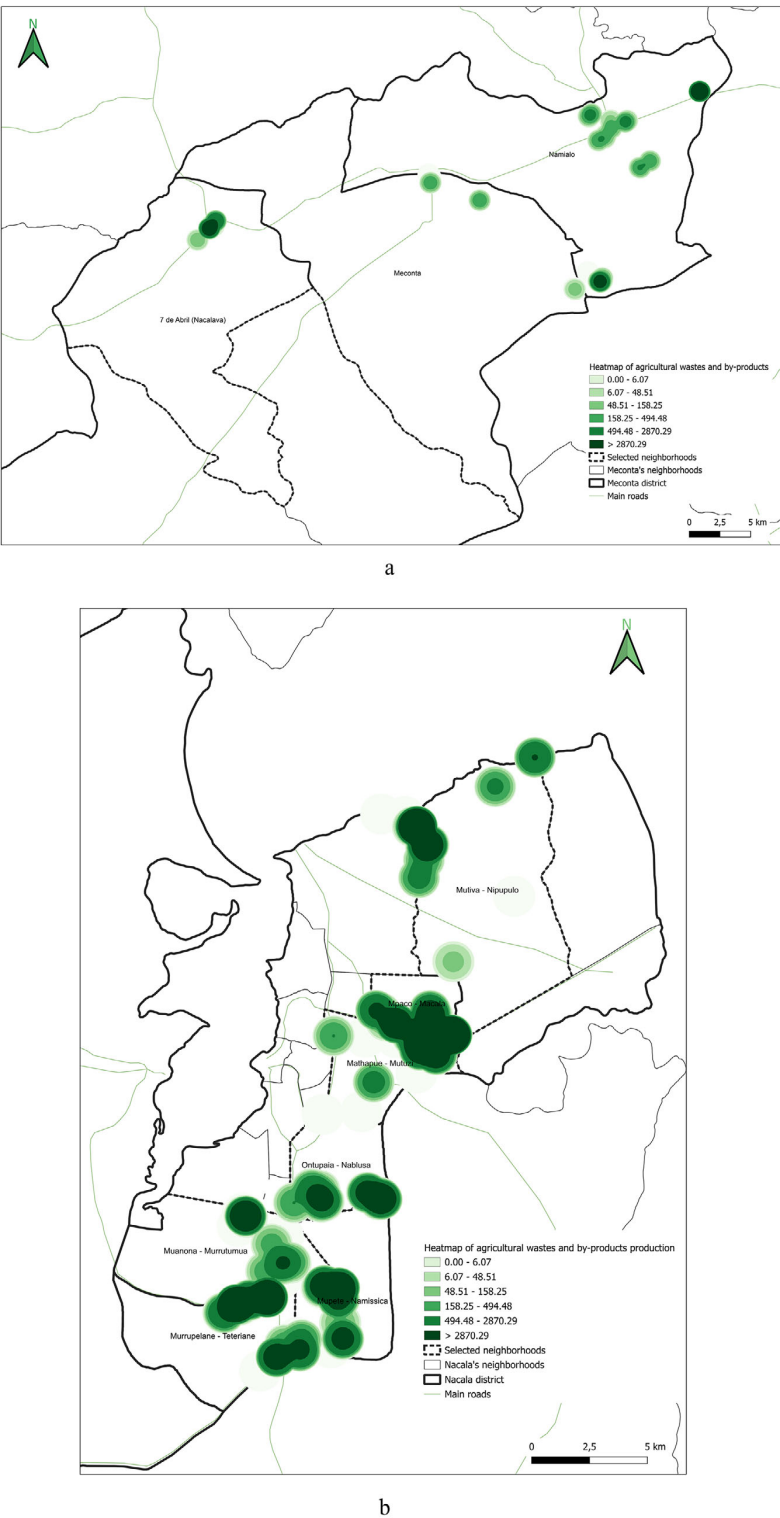
Heatmap of agricultural waste and by‐products availability in both Meconta (a) and Nacala (b) districts.

The heatmaps illustrating the spatial distribution of agricultural wastes and by‐products in the districts of Meconta and Nacala (Figure 21) form a central component of the territorial analysis. These visual representations are particularly valuable for identifying priority areas with high potential for bioenergy production, especially through the valorization of crop residues. By incorporating spatial data on land use patterns, crop typology, agricultural output, and waste generation, the heatmaps provide a spatially explicit understanding of bioresource availability, supporting informed decision‐making for the deployment of community‐scale, sustainable technologies.

In Meconta district, patterns of agricultural waste generation are localized yet significant. As illustrated in Figure 21a, the north‐eastern sector near Namialo and parts of the south‐eastern zone show the highest estimated residue outputs, ranging from 494.48 to 2870.29 tons. These areas are characterized by relatively intense agricultural activity and are considered strategic candidates for the implementation of bioresource recovery technologies.

In contrast, the central and southwestern areas, including neighborhoods such as 7 de Abril‐Nacalava, report consistently low residue volumes—between 0.00 and 6.07 tons. These low figures may stem from a combination of subsistence‐level agriculture, infrastructure limitations, or inefficiencies in the post‐harvest supply chain. Notably, some of these low‐output areas still maintain substantial cultivated land, suggesting issues such as land underutilization, low‐yield crops, or a disconnect between production and residue potential.

Figure 21b presents a more spatially complex picture in Nacala district, where higher agricultural intensity and larger cultivated surfaces produce broader patterns of bioresource availability. The southern and central zones display the highest concentrations of agricultural waste, exceeding 2870.29 tons. Key neighborhoods such as Teterranea, Namissica, and Mpaco emerge as bioresource hotspots, combining both expansive farmland and intensive farming practices.

Intermediate waste generation, ranging from 158.25 to 494.48 tons, is observed in areas such as Nablusa and Mutuzi. These zones may represent underutilized opportunities for future residue valorization initiatives. Meanwhile, northern sectors, including Mutiva, and peripheral neighborhoods in the west fall within the lowest waste generation category (0.00–48.51 tons), suggesting limited agricultural output or predominant subsistence farming practices.

A key insight from this analysis is that larger cultivated areas do not necessarily correlate with higher levels of agricultural waste production. This paradox can be explained by a variety of factors, including crop type and use (e.g., fresh vs. processed markets), farming techniques, and residue management practices. Furthermore, water use—a critical dimension of the water‐energy nexus—is influenced not only by output quantity, but also by local water availability, irrigation infrastructure, and efficiency of water application methods.

By leveraging geospatial visualization, it becomes possible to identify zones where a favorable balance exists between agricultural productivity, residue availability, and water demand. These are ideal target areas for the deployment of integrated water‐energy technologies, particularly those aligned with circular economy principles.

**FIGURE 22:**
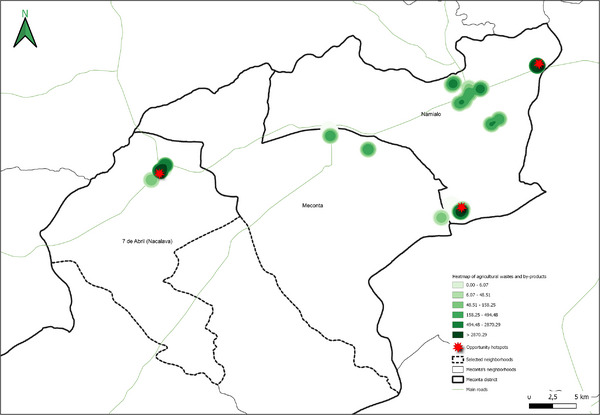
Heatmap of agricultural waste and by‐products availability in Meconta, with identification of opportunity hotspots.

In Meconta, three opportunity hotspots have been identified based on this integrated spatial analysis (Figure 22). These zones are distributed across distinct neighborhoods and exhibit sufficient agricultural waste availability combined with moderate water demands, making them prime candidates for community‐level technology deployment such as biogas production, composting, or multiresource hubs.

**FIGURE 23:**
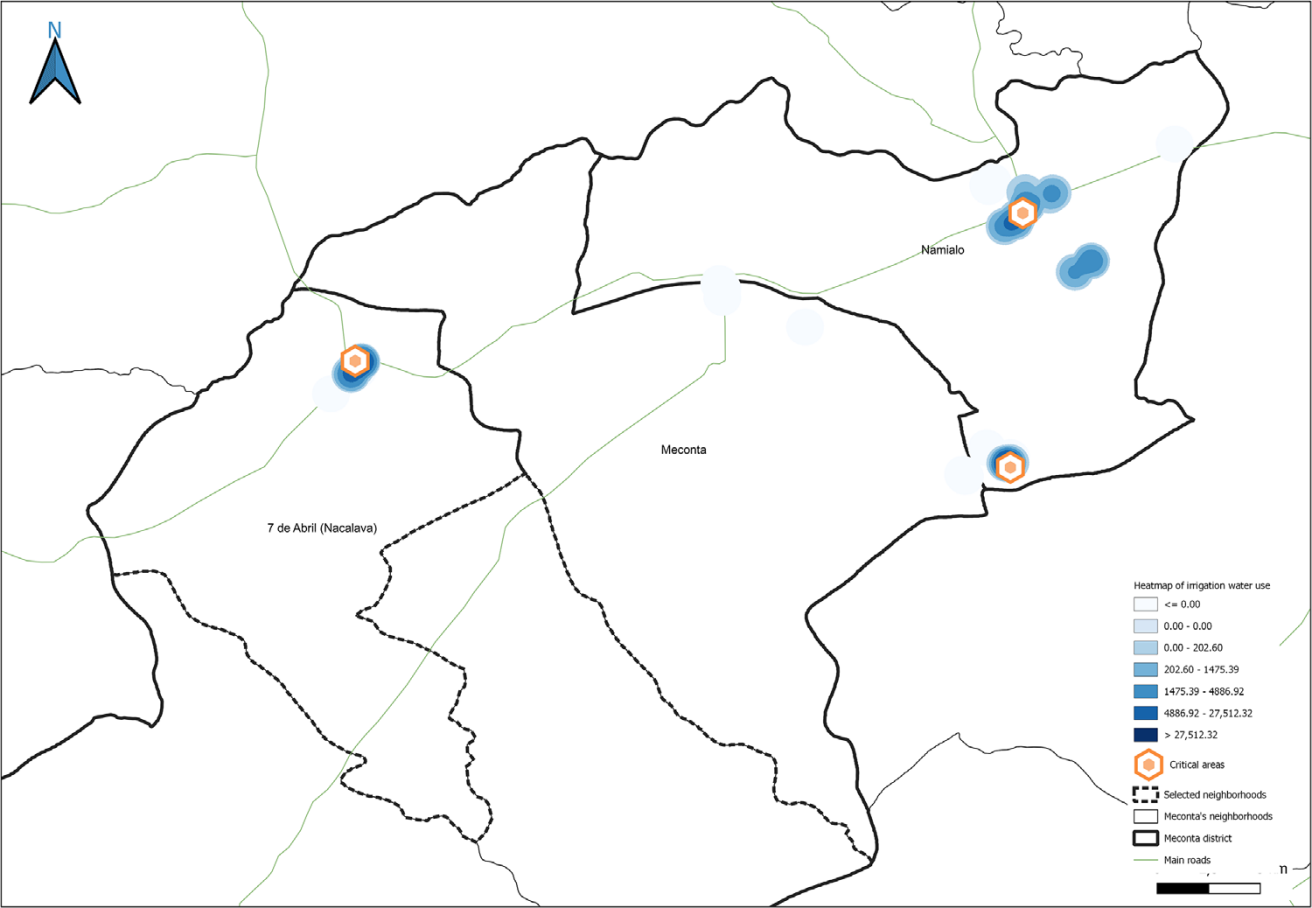
Heatmap showing irrigation water consumption in Meconta, identifying high‐demand critical areas.

**FIGURE 24:**
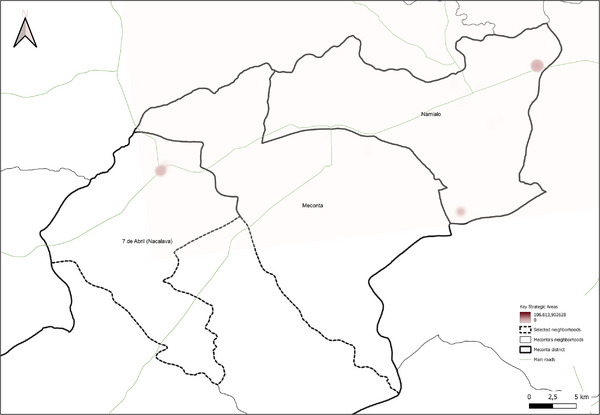
Strategic zones in Meconta for integrated water and bioresource management.

While Meconta offers promising areas for intervention, the analysis also reveals critical zones of concern, especially regarding high irrigation water consumption. In Figure 23 three zones are identified, where water demand is disproportionately high and may pose long‐term sustainability risks. Of particular interest is the westernmost area, which lies adjacent to a natural wetland. This location could potentially serve as an alternative water source, provided that appropriate and sustainable infrastructure is developed (Figure 24).

The spatial overlay of water consumption and waste availability helps define three priority zones for integrated resource management, where technological interventions could simultaneously address energy, water, and waste challenges.

**FIGURE 25:**
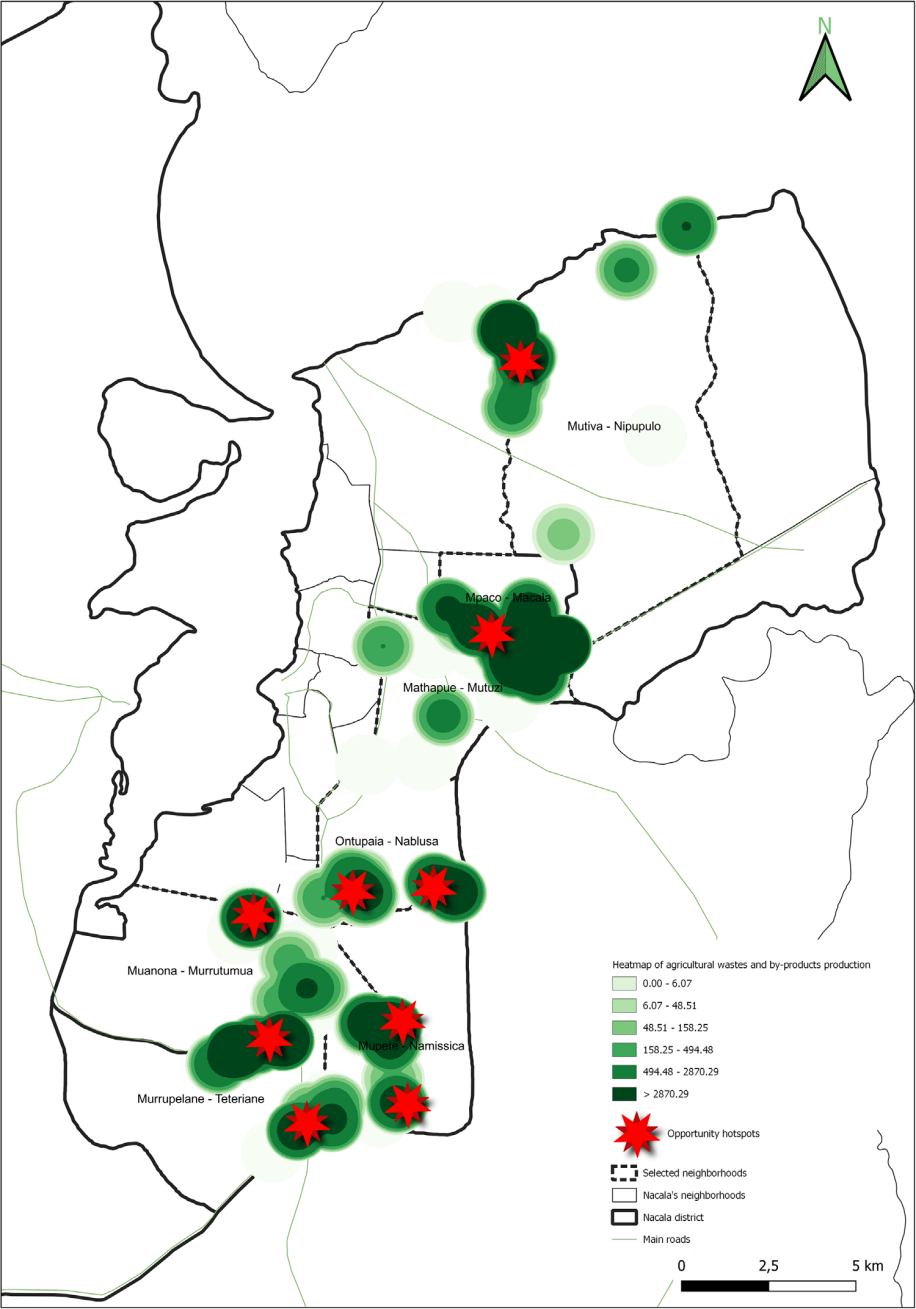
Heatmap of agricultural waste and by‐products availability in Nacala, with identification of opportunity hotspots.

Compared to Meconta, Nacala exhibits a more intensive agricultural landscape and a denser network of production zones. Figure 25 identifies nine high‐potential zones across seven neighborhoods with significant agricultural waste availability. These zones are well‐suited for bioenergy generation, organic fertilizer production, and other circular economy strategies.

**FIGURE 26:**
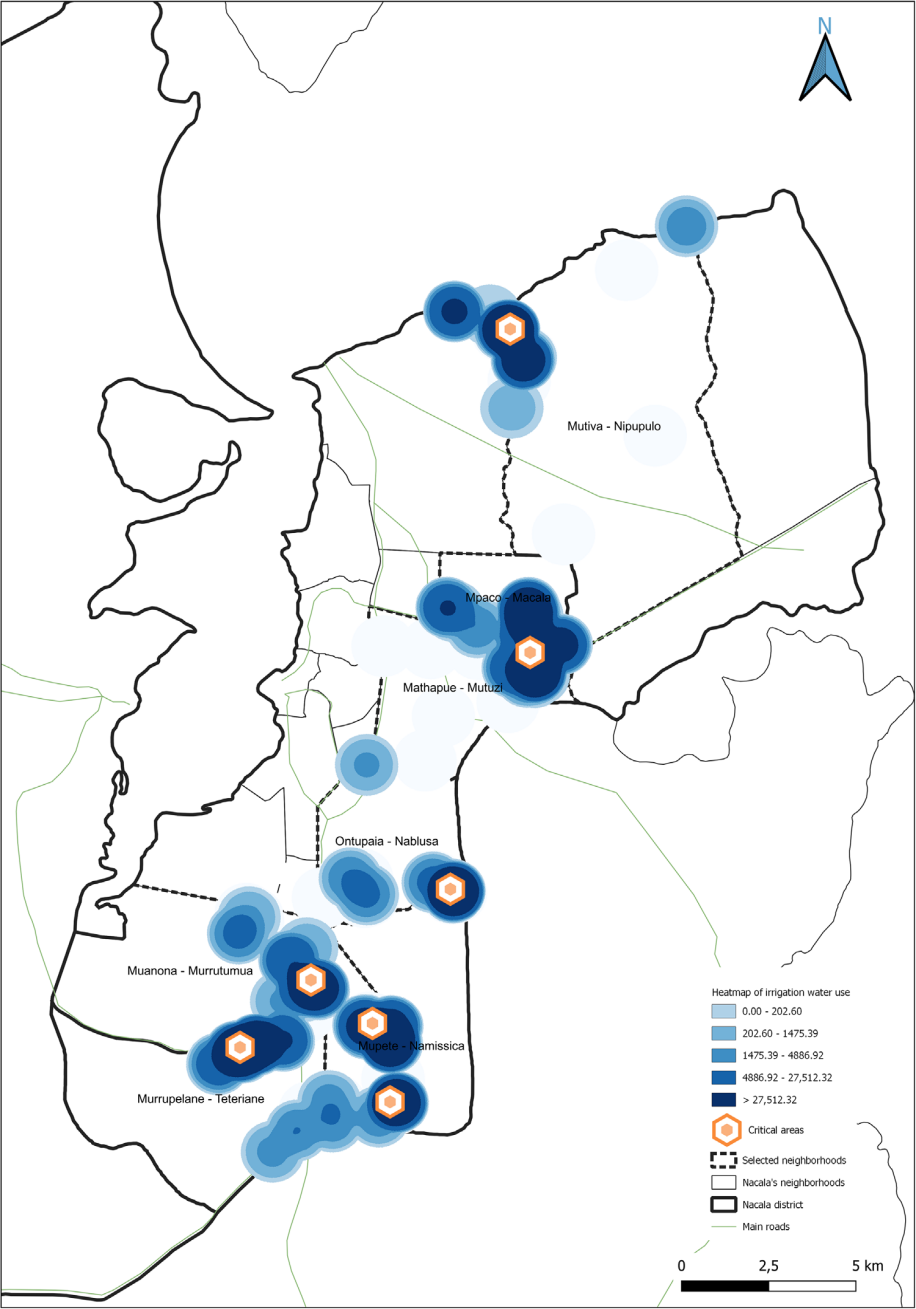
Irrigation water consumption heatmap in Nacala, highlighting critical high‐demand zones.

However, this potential is tempered by intensive irrigation practices. Figure 26 highlights seven zones under high water stress, underscoring the need for urgent resource planning to prevent depletion of local aquifers and surface water sources.

**FIGURE 27:**
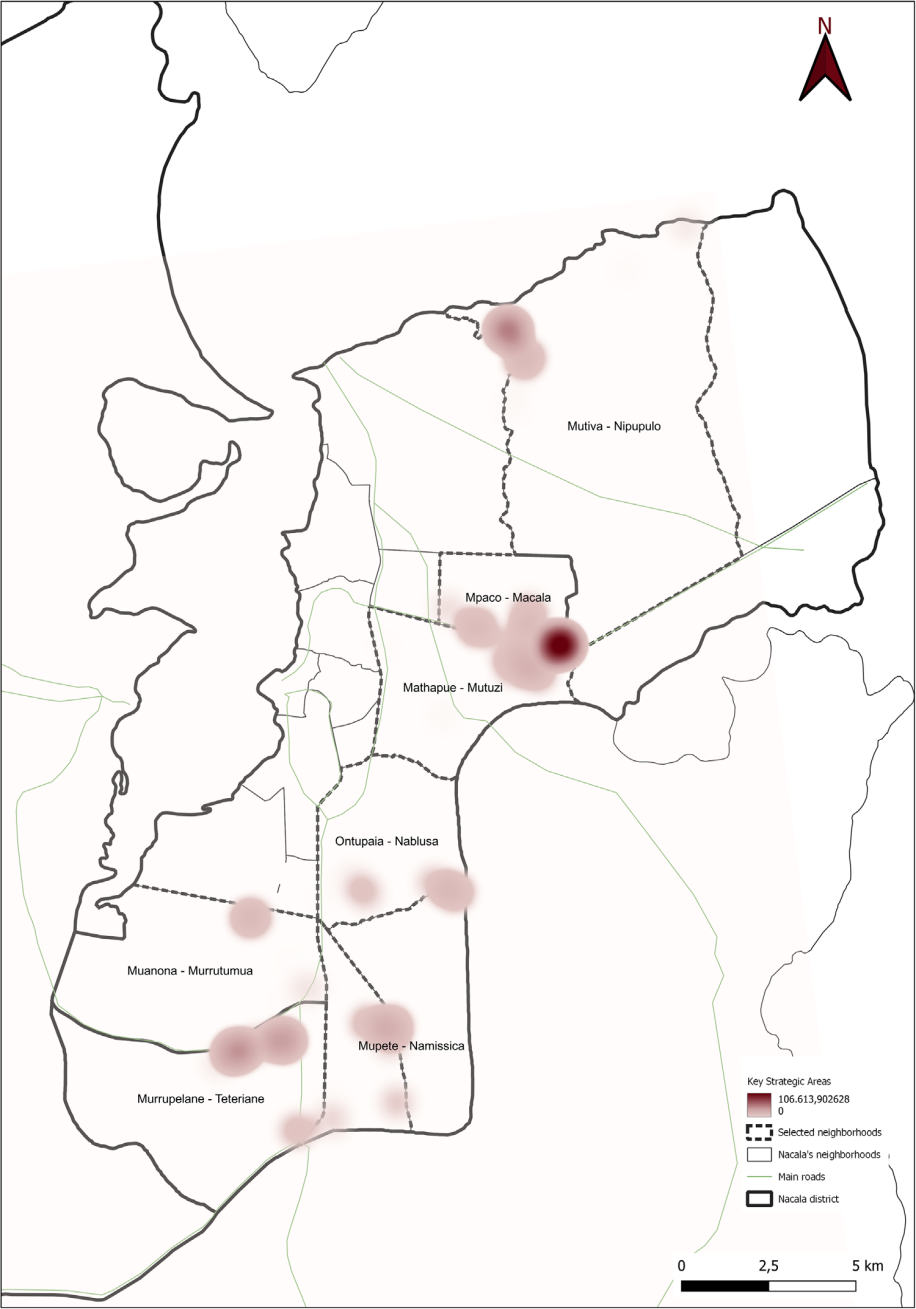
Strategic zones in Nacala identified for integrated water‐energy resource management.

The overlay in Figure 27 reveals that several zones are subject to both high bioresource availability and high‐water consumption. These dual‐priority areas are critical to future planning efforts. Effective interventions here could maximize energy output while addressing water conservation and climate resilience goals.

Importantly, while these findings are specific to Meconta and Nacala, the methodology and insights are generalizable to other rural districts and peri‐urban areas in Mozambique and across sub‐Saharan Africa. Similar patterns of biomass‐water mismatch and high‐value dual‐priority zones are likely to occur in regions facing comparable climatic, infrastructural, and socioeconomic challenges. This situates the study within the broader literature on sustainable agricultural waste management and circular economy applications, extending the practical relevance of GIS‐based participatory approaches for planning integrated water‐energy interventions [6, 13, 14]. By adapting the GIS‐based approach and participatory engagement model, planners in other districts can prioritize interventions, allocate resources efficiently, and replicate circular economy strategies in contexts with comparable challenges. This spatially driven analysis reaffirms the importance of multivariable geographic planning in advancing sustainable agriculture and supporting the energy transition in Mozambique. By integrating data on waste availability, land use intensity, and water stress, the heatmaps act as essential decision‐support tools for stakeholders, policymakers, and researchers.

In both Meconta and Nacala, zones of opportunity and concern have been mapped and analyzed. These insights enable more targeted interventions, helping to ensure that limited resources—such as water and biomass—are used efficiently and sustainably. The approach also highlights how integrating spatial analytics with local stakeholder knowledge can inform broader regional development strategies, supporting policy formulation that considers both local needs and systemic resource management across multiple districts. As the country moves toward more resilient rural development models, such tools will be instrumental in guiding the deployment of community‐based, integrated technologies that align with both climate goals and local development priorities.

## 4 Conclusions

This study presents an integrated and participatory methodology to address pressing water and energy challenges in rural Mozambique through the spatial valorization of agricultural waste. Focusing on the districts of Meconta and Nacala in Nampula Province, the research highlights the underutilized potential of agricultural residues as a catalyst for sustainable resource management, expanded energy access, and environmental regeneration.

By combining GIS tools with field data and participatory engagement, the study delineates opportunity zones and critical intervention areas, generating spatially explicit heatmaps and overlays that serve as decision‐support tools for assessing biomass availability, irrigation demand, and land‐use intensity. In Meconta, three opportunity hotspots were identified where moderate water demands intersect with significant agricultural residue availability. These areas present strong potential for deploying community‐scale bioenergy solutions such as anaerobic digestion, composting, or integrated multiresource hubs. At the same time, spatial analysis revealed zones of disproportionately high irrigation water consumption—especially near ecologically sensitive areas such as natural wetlands—underscoring the urgency for sustainable water infrastructure and integrated management strategies.

Nacala's landscape, characterized by dense agricultural activity, offers even greater potential for agricultural waste valorization, with nine high‐biomass zones mapped across seven neighborhoods. However, this potential is counterbalanced by high irrigation demands in several of these same areas. The intersection of high biomass availability and water stress defines dual‐priority zones, highlighting areas where carefully coordinated interventions can maximize impact across the water–energy–food (WEF) nexus.

A key insight from the analysis is that agricultural land area alone does not predict biomass availability. Instead, factors such as crop type, postharvest practices, and accessibility strongly influence the quantity and usability of agricultural residues. This reinforces the value of localized, data‐informed approaches and illustrates the novelty of integrating spatial and participatory methods for planning resource‐efficient interventions.

The integration of spatial analytics with qualitative fieldwork—such as interviews with farmers, cooperatives, and local institutions—ensured the robustness of the results. This bottom‐up methodology, involving rural communities and stakeholders, increases the likelihood of adoption and long‐term success, particularly in governance environments marked by institutional fragmentation and limited rural infrastructure.

The study provides a scalable and replicable GIS‐based framework that bridges technical and social dimensions, supporting circular economy principles and Mozambique's sustainability agenda. By repositioning agricultural waste as a resource rather than a liability, the framework enables a dual transition toward climate resilience and inclusive rural development. The deployment of low‐cost, decentralized technologies in identified zones offers tangible pathways to reduce deforestation, improve food security, and expand energy access while respecting local socioenvironmental constraints. Sustained adoption of these solutions requires active involvement of local and national institutions. District authorities can facilitate community engagement, provide technical support, and integrate waste valorization into rural development plans, while national ministries responsible for agriculture, energy, and water can establish enabling policies, allocate incentives, and strengthen extension services. Funding mechanisms from national budgets and international cooperation programs can support pilot projects and replication in other provinces.

Key policy recommendations include (i) capacity‐building to enhance cross‐sectoral coordination across water, energy, and agriculture; (ii) establishment of multi‐level governance platforms to align local interventions with national strategies; and (iii) creation of regulatory frameworks incentivizing private sector and cooperative participation in waste valorization. Adaptive governance mechanisms are essential to manage trade‐offs and synergies at local levels, ensuring interventions are equitable, efficient, and resilient.

This research contributes to the literature by demonstrating how a GIS‐based, participatory approach can integrate technical, socioeconomic, and governance considerations to inform sustainable rural development. Spatially driven, participatory planning is therefore a cornerstone for transforming agricultural waste into opportunities, providing actionable insights for policymakers, NGOs, and development actors across Mozambique and other sub‐Saharan contexts.

## Funding

This research was carried out within the project “GREENFLOW: Sustainable Solutions for Water‐Energy Nexus” ‐ GLOBAL SOUTH 2024, project code “GLOBAL_SOUTH_2024_DISTAL, CUP J15C24000120005,” coordinated by Prof. Francesca Valenti and funded by Alma Mater Studiorum ‐ University of Bologna.

## Conflicts of Interest

The authors declare no conflict of interest.

## Supporting information




**Supporting Information File 1**: gch270067‐sup‐0001‐SuppMat.docx

## Data Availability

The data that support the findings of this study are available from the corresponding author upon reasonable request.
