# Supplementary material for: Spatial Analysis of Agricultural Waste and By‐Products to Tackle the Water–Energy Nexus in Rural Mozambique
Source: Glob Chall. 2025 Nov 29;10(1):e00339. doi: 10.1002/gch2.202500339 (PMC12776009; doi:10.1002/gch2.202500339)
Supplement: Supplementary file 1 — Supporting Information File 1: gch270067‐sup‐0001‐SuppMat.docx [file GCH2-10-e00339-s001.docx]

# Spatial Analysis of Agricultural Waste and By-products to Tackle the Water-Energy Nexus in Rural Mozambique

*Mancuso Giuseppe, Morini Valentina, Martinez Gonzalo A., Toscano Attilio, Valenti Francesca**

*Mancuso G., Morini V., Toscano A., Valenti F.:* Department of Agricultural and Food Sciences (DISTAL), Alma Mater Studiorum - University of Bologna, viale Fanin 50, 40127 Bologna, Italy

*Martinez G.A.:* Department of Civil, Chemical, Environmental and Materials Engineering (DICAM), University of Bologna, via Terracini 28, I-40131 Bologna, Italy

* Corresponding author: francesca.valenti9@unibo.it

***Questionnaire S1***

*Informações sobre a empresa/cooperativa/agricultor individual*

1. **Distrito:**

□ Nacala

□Meconta

1. **Nome do Bairro:**

□ Mpaco

□ Mutuzi

□ Murrutumua

□ Namissica

□ Teterranea

□ Nablusa (Waleite)

□ Mutiva

□ Vila Sede de Meconta

□ Areial-Namialo

□ Viera-Namialo

□7 de abril-Nacavala

1. Localização (Coordenada GPS)___________________
2. **Tipo de Empresa:**

□ Empresa Agrícola

□ Cooperativa

□ Associação de vários agricultores

□ Empresário Agricultor

1. Área Total da Empresa [quantidade em m²] _______________
2. Área para Agricultura [quantidade em m²] _______________
3. Área para Pecuária [quantidade em m²] _______________
4. **Pratica a pecuária?**

□ Sim

□ Não

- Se sim, tipo de espécies de animais criados:

□ Bovinos [número]_________ excrementos [quantidade anual em kg] _________
□ Suínos [número]_________ excrementos [quantidade anual em kg] _________
□ Caprinos [número]______ excrementos [quantidade anual em kg] _________
□ Galinhas [número]______ excrementos [quantidade anual em kg] _________
□ Outros [especificar]__________ [número]_____ excrementos [quantidade anual em kg] ______

1. **Pratica a agricultura?**

□ Sim

□ Não

1. **Se sim, pratica monocultura ou policultura**

□ Monocoltura

□ Policoltura

1. **Pratica consorciação de culturas?**

□ Sim

□ Não

1. **Que culturas cultiva?**

**Tipo de cultura 1 [nome]________________**

***TIPO DE CULTURA 1:***

- Realiza mais de um ciclo cultural por ano para esta cultura?

□ Não

□ Sim

- Se sim, quantos?

________________

-Ciclo 1: Estacionalidade das culturas (períodos de plantio e colheita):

□ Período de plantio [meses] _____________

□ Período de colheita [meses] _____________

-Ciclo 2: Estacionalidade das culturas (períodos de plantio e colheita):

□ Período de plantio [meses] _____________

□ Período de colheita [meses] _____________

-Área agrícola utilizada para esta cultura:

□ Quantitade em ha/m² ______________

-Realiza irrigação para esta cultura?

□ Sim

□ Não

-Métodos utilizados para irrigação

□ Manual
□ Mecânico
□ Misto

-Se mecânico ou misto, qual tipo?

□ Escorrimento
□ Pressão
□ Gota a gota (microirrigação)
□ Outro [especificar] ______________

-**Quantidade de água utilizada para irrigação:**

□ Quantidade em litros______________

-Produção total anual para a cultura

□Quantidade em kg ______________

-Técnicas de colheita:

□ Manuais
□ Mecânicas

-Se mecânicas, com quais equipamentos?

­­­­­­­_____________________________

-Gestão dos produtos pós-colheita:

□ Vendido fresco [quantidade anual em kg] ______________
□ Transformado [quantidade anual em kg] ______________
□ Outro [especificar] ______________ [quantidade anual em kg] ______________

-Tipo e quantidade de resíduos/subprodutos agrícolas e animais (palha, cascas, resíduos de poda, excrementos, etc.) obtidos e utilização/tratamento atual:

- - Resíduos e subprodutos [nome] ______________ [quantidade anual em kg] ______________
    □ Queimado □ Compostado □ Usado como fertilizante □ Outro [especificar] ______________
  - Resíduos e subprodutos [nome] ______________ [quantidade anual em kg] ______________
    □ Queimado □ Compostado □ Usado como fertilizante □ Outro [especificar] ______________
  - Resíduos e subprodutos [nome] ______________ [quantidade anual em kg] ______________
    □ Queimado □ Compostado □ Usado como fertilizante □ Outro [especificar] ______________

**Tipo de cultura 2 [nome]________________**

TIPO DE CULTURA 2:

- Realiza mais de um ciclo cultural por ano para esta cultura?

□ Não

□ Sim

- Se sim, quantos?

________________

-Ciclo 1: Estacionalidade das culturas (períodos de plantio e colheita):

□ Período de plantio [meses] _____________

□ Período de colheita [meses] _____________

-Ciclo 2: Estacionalidade das culturas (períodos de plantio e colheita):

□ Período de plantio [meses] _____________

□ Período de colheita [meses] _____________

-Área agrícola utilizada para esta cultura:

□ Quantitade em ha/m² ______________

-Realiza irrigação para esta cultura?

□ Sim

□ Não

-Métodos utilizados para irrigação

□ Manual
□ Mecânico
□ Misto

-Se mecânico ou misto, qual tipo?

□ Escorrimento
□ Pressão
□ Gota a gota (microirrigação)
□ Outro [especificar] ______________

-**Quantidade de água utilizada para irrigação:**

□ Quantidade em litros______________

-Produção total anual para a cultura

□Quantidade em kg/toneladas ______________

-Técnicas de colheita:

□ Manuais
□ Mecânicas

-Se mecânicas, com quais equipamentos?

­­­­­­­_____________________________

-Gestão dos produtos pós-colheita:

□ Vendido fresco [quantidade anual em kg] ______________
□ Transformado [quantidade anual em kg] ______________
□ Outro [especificar] ______________ [quantidade anual em kg] ______________

-Tipo e quantidade de resíduos/subprodutos agrícolas e animais (palha, cascas, resíduos de poda, excrementos, etc.) obtidos e utilização/tratamento atual:

- - Resíduos e subprodutos [nome] ______________ [quantidade anual em kg] ______________
    □ Queimado □ Compostado □ Usado como fertilizante □ Outro [especificar] ______________
  - Resíduos e subprodutos [nome] ______________ [quantidade anual em kg] ______________
    □ Queimado □ Compostado □ Usado como fertilizante □ Outro [especificar] ______________
  - Resíduos e subprodutos [nome] ______________ [quantidade anual em kg] ______________
    □ Queimado □ Compostado □ Usado como fertilizante □ Outro [especificar] ______________

**Tipo de cultura 3 [nome]________________**

***TIPO DE CULTURA 3:***

- Realiza mais de um ciclo cultural por ano para esta cultura?

□ Não

□ Sim

- Se sim, quantos?

________________

-Ciclo 1: Estacionalidade das culturas (períodos de plantio e colheita):

□ Período de plantio [meses] _____________

□ Período de colheita [meses] _____________

-Ciclo 2: Estacionalidade das culturas (períodos de plantio e colheita):

□ Período de plantio [meses] _____________

□ Período de colheita [meses] _____________

-Área agrícola utilizada para esta cultura:

□ Quantitade em ha/m² ______________

-Realiza irrigação para esta cultura?

□ Sim

□ Não

-Métodos utilizados para irrigação

□ Manual
□ Mecânico
□ Misto

-Se mecânico ou misto, qual tipo?

□ Escorrimento
□ Pressão
□ Gota a gota (microirrigação)
□ Outro [especificar] ______________

-**Quantidade de água utilizada para irrigação:**

□ Quantidade em litros______________

-Produção total anual para a cultura

□Quantidade em kg/toneladas ______________

-Técnicas de colheita:

□ Manuais
□ Mecânicas

-Se mecânicas, com quais equipamentos?

­­­­­­­_____________________________

-Gestão dos produtos pós-colheita:

□ Vendido fresco [quantidade anual em kg] ______________
□ Transformado [quantidade anual em kg] ______________
□ Outro [especificar] ______________ [quantidade anual em kg] ______________

-Tipo e quantidade de resíduos/subprodutos agrícolas e animais (palha, cascas, resíduos de poda, excrementos, etc.) obtidos e utilização/tratamento atual:

- - Resíduos e subprodutos [nome] ______________ [quantidade anual em kg] ______________
    □ Queimado □ Compostado □ Usado como fertilizante □ Outro [especificar] ______________
  - Resíduos e subprodutos [nome] ______________ [quantidade anual em kg] ______________
    □ Queimado □ Compostado □ Usado como fertilizante □ Outro [especificar] ______________
  - Resíduos e subprodutos [nome] ______________ [quantidade anual em kg] ______________
    □ Queimado □ Compostado □ Usado como fertilizante □ Outro [especificar] ______________

**Tipo de cultura 4 [nome]________________**

***TIPO DE CULTURA 4:***

- Realiza mais de um ciclo cultural por ano para esta cultura?

□ Não

□ Sim

- Se sim, quantos?

________________

-Ciclo 1: Estacionalidade das culturas (períodos de plantio e colheita):

□ Período de plantio [meses] _____________

□ Período de colheita [meses] _____________

-Ciclo 2: Estacionalidade das culturas (períodos de plantio e colheita):

□ Período de plantio [meses] _____________

□ Período de colheita [meses] _____________

-Área agrícola utilizada para esta cultura:

□ Quantitade em ha/m² ______________

-Realiza irrigação para esta cultura?

□ Sim

□ Não

-Métodos utilizados para irrigação

□ Manual
□ Mecânico
□ Misto

-Se mecânico ou misto, qual tipo?

□ Escorrimento
□ Pressão
□ Gota a gota (microirrigação)
□ Outro [especificar] ______________

-**Quantidade de água utilizada para irrigação:**

□ Quantidade em litros______________

-Produção total anual para a cultura

□Quantidade em kg/toneladas ______________

-Técnicas de colheita:

□ Manuais
□ Mecânicas

-Se mecânicas, com quais equipamentos?

­­­­­­­_____________________________

-Gestão dos produtos pós-colheita:

□ Vendido fresco [quantidade anual em kg] ______________
□ Transformado [quantidade anual em kg] ______________
□ Outro [especificar] ______________ [quantidade anual em kg] ______________

-Tipo e quantidade de resíduos/subprodutos agrícolas e animais (palha, cascas, resíduos de poda, excrementos, etc.) obtidos e utilização/tratamento atual:

- - Resíduos e subprodutos [nome] ______________ [quantidade anual em kg] ______________
    □ Queimado □ Compostado □ Usado como fertilizante □ Outro [especificar] ______________
  - Resíduos e subprodutos [nome] ______________ [quantidade anual em kg] ______________
    □ Queimado □ Compostado □ Usado como fertilizante □ Outro [especificar] ______________
  - Resíduos e subprodutos [nome] ______________ [quantidade anual em kg] ______________
    □ Queimado □ Compostado □ Usado como fertilizante □ Outro [especificar] ______________

**Tipo de cultura 5 [nome]________________**

***TIPO DE CULTURA 5:***

- Realiza mais de um ciclo cultural por ano para esta cultura?

□ Não

□ Sim

- Se sim, quantos?

________________

-Ciclo 1: Estacionalidade das culturas (períodos de plantio e colheita):

□ Período de plantio [meses] _____________

□ Período de colheita [meses] _____________

-Ciclo 2: Estacionalidade das culturas (períodos de plantio e colheita):

□ Período de plantio [meses] _____________

□ Período de colheita [meses] _____________

-Área agrícola utilizada para esta cultura:

□ Quantitade em ha/m² ______________

-Realiza irrigação para esta cultura?

□ Sim

□ Não

-Métodos utilizados para irrigação

□ Manual
□ Mecânico
□ Misto

-Se mecânico ou misto, qual tipo?

□ Escorrimento
□ Pressão
□ Gota a gota (microirrigação)
□ Outro [especificar] ______________

-**Quantidade de água utilizada para irrigação:**

□ Quantidade em litros______________

-Produção total anual para a cultura

□Quantidade em kg/toneladas ______________

-Técnicas de colheita:

□ Manuais
□ Mecânicas

-Se mecânicas, com quais equipamentos?

­­­­­­­_____________________________

-Gestão dos produtos pós-colheita:

□ Vendido fresco [quantidade anual em kg] ______________
□ Transformado [quantidade anual em kg] ______________
□ Outro [especificar] ______________ [quantidade anual em kg] ______________

-Tipo e quantidade de resíduos/subprodutos agrícolas e animais (palha, cascas, resíduos de poda, excrementos, etc.) obtidos e utilização/tratamento atual:

- - Resíduos e subprodutos [nome] ______________ [quantidade anual em kg] ______________
    □ Queimado □ Compostado □ Usado como fertilizante □ Outro [especificar] ______________
  - Resíduos e subprodutos [nome] ______________ [quantidade anual em kg] ______________
    □ Queimado □ Compostado □ Usado como fertilizante □ Outro [especificar] ______________
  - Resíduos e subprodutos [nome] ______________ [quantidade anual em kg] ______________
    □ Queimado □ Compostado □ Usado como fertilizante □ Outro [especificar] ______________

**Tipo de cultura 6 [nome]________________**

***TIPO DE CULTURA 6:***

- Realiza mais de um ciclo cultural por ano para esta cultura?

□ Não

□ Sim

- Se sim, quantos?

________________

-Ciclo 1: Estacionalidade das culturas (períodos de plantio e colheita):

□ Período de plantio [meses] _____________

□ Período de colheita [meses] _____________

-Ciclo 2: Estacionalidade das culturas (períodos de plantio e colheita):

□ Período de plantio [meses] _____________

□ Período de colheita [meses] _____________

-Área agrícola utilizada para esta cultura:

□ Quantitade em ha/m² ______________

-Realiza irrigação para esta cultura?

□ Sim

□ Não

-Métodos utilizados para irrigação

□ Manual
□ Mecânico
□ Misto

-Se mecânico ou misto, qual tipo?

□ Escorrimento
□ Pressão
□ Gota a gota (microirrigação)
□ Outro [especificar] ______________

-**Quantidade de água utilizada para irrigação:**

□ Quantidade em litros______________

-Produção total anual para a cultura

□Quantidade em kg/toneladas ______________

-Técnicas de colheita:

□ Manuais
□ Mecânicas

-Se mecânicas, com quais equipamentos?

­­­­­­­_____________________________

-Gestão dos produtos pós-colheita:

□ Vendido fresco [quantidade anual em kg] ______________
□ Transformado [quantidade anual em kg] ______________
□ Outro [especificar] ______________ [quantidade anual em kg] ______________

-Tipo e quantidade de resíduos/subprodutos agrícolas e animais (palha, cascas, resíduos de poda, excrementos, etc.) obtidos e utilização/tratamento atual:

- - Resíduos e subprodutos [nome] ______________ [quantidade anual em kg] ______________
    □ Queimado □ Compostado □ Usado como fertilizante □ Outro [especificar] ______________
  - Resíduos e subprodutos [nome] ______________ [quantidade anual em kg] ______________
    □ Queimado □ Compostado □ Usado como fertilizante □ Outro [especificar] ______________
  - Resíduos e subprodutos [nome] ______________ [quantidade anual em kg] ______________
    □ Queimado □ Compostado □ Usado como fertilizante □ Outro [especificar] ______________

**Tipo de cultura 7 [nome]________________**

***TIPO DE CULTURA 7:***

- Realiza mais de um ciclo cultural por ano para esta cultura?

□ Não

□ Sim

- Se sim, quantos?

________________

-Ciclo 1: Estacionalidade das culturas (períodos de plantio e colheita):

□ Período de plantio [meses] _____________

□ Período de colheita [meses] _____________

-Ciclo 2: Estacionalidade das culturas (períodos de plantio e colheita):

□ Período de plantio [meses] _____________

□ Período de colheita [meses] _____________

-Área agrícola utilizada para esta cultura:

□ Quantitade em ha/m² ______________

-Realiza irrigação para esta cultura?

□ Sim

□ Não

-Métodos utilizados para irrigação

□ Manual
□ Mecânico
□ Misto

-Se mecânico ou misto, qual tipo?

□ Escorrimento
□ Pressão
□ Gota a gota (microirrigação)
□ Outro [especificar] ______________

-**Quantidade de água utilizada para irrigação:**

□ Quantidade em litros______________

-Produção total anual para a cultura

□Quantidade em kg/toneladas ______________

-Técnicas de colheita:

□ Manuais
□ Mecânicas

-Se mecânicas, com quais equipamentos?

­­­­­­­_____________________________

-Gestão dos produtos pós-colheita:

□ Vendido fresco [quantidade anual em kg] ______________
□ Transformado [quantidade anual em kg] ______________
□ Outro [especificar] ______________ [quantidade anual em kg] ______________

-Tipo e quantidade de resíduos/subprodutos agrícolas e animais (palha, cascas, resíduos de poda, excrementos, etc.) obtidos e utilização/tratamento atual:

- - Resíduos e subprodutos [nome] ______________ [quantidade anual em kg] ______________
    □ Queimado □ Compostado □ Usado como fertilizante □ Outro [especificar] ______________
  - Resíduos e subprodutos [nome] ______________ [quantidade anual em kg] ______________
    □ Queimado □ Compostado □ Usado como fertilizante □ Outro [especificar] ______________
  - Resíduos e subprodutos [nome] ______________ [quantidade anual em kg] ______________
    □ Queimado □ Compostado □ Usado como fertilizante □ Outro [especificar] ______________

**Tipo de cultura 8 [nome]________________**

***TIPO DE CULTURA 8:***

- Realiza mais de um ciclo cultural por ano para esta cultura?

□ Não

□ Sim

- Se sim, quantos?

________________

-Ciclo 1: Estacionalidade das culturas (períodos de plantio e colheita):

□ Período de plantio [meses] _____________

□ Período de colheita [meses] _____________

-Ciclo 2: Estacionalidade das culturas (períodos de plantio e colheita):

□ Período de plantio [meses] _____________

□ Período de colheita [meses] _____________

-Área agrícola utilizada para esta cultura:

□ Quantitade em ha/m² ______________

-Realiza irrigação para esta cultura?

□ Sim

□ Não

-Métodos utilizados para irrigação

□ Manual
□ Mecânico
□ Misto

-Se mecânico ou misto, qual tipo?

□ Escorrimento
□ Pressão
□ Gota a gota (microirrigação)
□ Outro [especificar] ______________

-**Quantidade de água utilizada para irrigação:**

□ Quantidade em litros______________

-Produção total anual para a cultura

□Quantidade em kg/toneladas ______________

-Técnicas de colheita:

□ Manuais
□ Mecânicas

-Se mecânicas, com quais equipamentos?

­­­­­­­_____________________________

-Gestão dos produtos pós-colheita:

□ Vendido fresco [quantidade anual em kg] ______________
□ Transformado [quantidade anual em kg] ______________
□ Outro [especificar] ______________ [quantidade anual em kg] ______________

-Tipo e quantidade de resíduos/subprodutos agrícolas e animais (palha, cascas, resíduos de poda, excrementos, etc.) obtidos e utilização/tratamento atual:

- - Resíduos e subprodutos [nome] ______________ [quantidade anual em kg] ______________
    □ Queimado □ Compostado □ Usado como fertilizante □ Outro [especificar] ______________
  - Resíduos e subprodutos [nome] ______________ [quantidade anual em kg] ______________
    □ Queimado □ Compostado □ Usado como fertilizante □ Outro [especificar] ______________
  - Resíduos e subprodutos [nome] ______________ [quantidade anual em kg] ______________
    □ Queimado □ Compostado □ Usado como fertilizante □ Outro [especificar] ______________

***Parte Água:***

1. Tipos de fontes de água utilizadas:

□ Rio/ Torrentes /Canais
□ Lago
□ Poço
□ Fonte
□ Tanques de coleta de água da chuva

• Tipo ______________________

• Capacidade (m3/l) ______________
□ Outro [especificar] ______________________

2. Distância da fonte de água Metri : ______________

***Parte Energia:***

1.Utiliza energia pare irrigação ?

□ Sim

□Não

2.Tipo e quantidade de energia utilizada para actividades agrícolas:

□ Irrigação: tipo de energia ______________ [quantidade anual Kw] ______________
□ Transformação/conservação de produtos locais: tipo de energia ______________ [quantidade anual Kw] ______________
□ Outras actividades [especificar] ____________ tipo de energia ______________ [quantidade anual Kw] ______________

3. Existem instalações para:

□ Bioenergia
 □ Agri-voltaico
 □ Outro [especificar] ______________

***Parte Maquinaria Agrícola:***

1.Como realiza as práticas agrícolas?

□ Manualmente
□ Mecânico com uso de máquinas
□ Misto

- Se mecânico, quais são os tratores e/ou máquinas utilizadas:
  - Máquina 1 ______________
    Tipo ______________
    Modelo ______________
    Combustível: □ Diesel □ Gasolina □ Outro [especificar] ______________
    Consumo anual de combustível [quantidade em litros] ______________
  - Máquina 2______________
    Tipo ______________
    Modelo ______________
    Combustível: □ Diesel □ Gasolina □ Outro [especificar] ______________
    Consumo anual de combustível [quantidade em litros] ______________
  - Máquina 3 ______________
    Tipo ______________
    Modelo ______________
    Combustível: □ Diesel □ Gasolina □ Outro [especificar] ______________
    Consumo anual de combustível [quantidade em litros] ______________
  - Máquina 4 ______________
    Tipo ______________
    Modelo ______________
    Combustível: □ Diesel □ Gasolina □ Outro [especificar] ______________
    Consumo anual de combustível [quantidade em litros] ______________

***Questionnaire S2***

*Informações sobre a empresas de transformação/mercados*

**1) Distrito:**
□ Nacala
□ Meconta

**2) Nome do Bairro:**
□ Mpaco
□ Mutuzi
□ Murrutumua
□ Namissica
□ Teterranea
□ Nablusa (Waleite)
□ Mutiva
□ Vila Sede de Meconta
□ Areial-Namialo
□ Viera-Namialo
□ 7 de Abril-Nacavala

**3) Localização (GPS):** ___________________

**4) Tipologia de transformação agro-alimentar realizada:** ___________________

**5) Tipologia de produtos transformados:**

**Nome 1:** ___________________
**Nome 2:** ___________________
**Nome 3:** ___________________
**Nome 4:** ___________________
**Nome 5:** ___________________
**Nome 6:** ___________________

**6)**

**Tipo de produto transformado 1 [nome]:** ________________

**PRODUTO TRANSFORMADO 1:**

**A)**
Quantidade anual em Kg? ______________
Tem resíduos e subprodutos da transformação?
□ Sim
□ Não
Se sim, quais são? [nome 1] ________ [quantidade anual Kg] ________
O que faz com esses resíduos?
□ **não reutilizo**
a. como os descarta? _________
b. em que quantidade anual (kg)? __________
□ **reutilizo**
□ **faço um tratamento para valorizá-los e reutilizá-los para o autoconsumo**
a. que tipo de tratamento faz? _________
b. em que quantidade anual? _________
c. se não for na totalidade, o que faz e como descarta o resto? ______________
□ **faço um tratamento para valorizá-los e vendê-los**
a. que tipo de tratamento faz? _________
b. em que quantidade anual? _________
c. se não for na totalidade, o que faz e como descarta o resto? ______________
**□ Ambas: tratamento para autoconsumo e venda:**
a. que tipo de tratamento faz? _________
b. em que quantidade anual? _________
c. quantidade para autoconsumo _________
d. quantidade para venda _________
e. se não for na totalidade, o que faz e como descarta o resto? ______________

**B)** Consumos anuais do processo de transformação PRODUTO 1:
a. Energia Eléctrica [quantidade anual em KW] ______
b. Energia Térmica via Caldeira
□ lenha [quantidade anual em Kg] ______
□ biomassa [quantidade anual em Kg] ______
c. Água [quantidade anual m³/litros] ______

**C)** Descreva as fases do processo de transformação PRODUTO 1:
Fase 1 [nome] ________________
Fase 2 [nome] ________________
Fase 3 [nome] ________________
Fase 4 [nome] ________________
Fase 5 [nome] ________________
Fase 6 [nome] ________________
Fase 7 [nome] ________________
Fase 8 [nome] ________________

**D)** Descreva o processo de transformação PRODUTO 1:

___________________________________________________________________________________________________________________________________________________________________________________________________________________________________________________________________________________________________________________________________________________________

**Tipo de produto transformado 2 [nome]:** ________________

**PRODUTO TRANSFORMADO 2:**

**A)**
Quantidade anual em Kg? ______________
Tem resíduos e subprodutos da transformação?
□ Sim
□ Não
Se sim, quais são? [nome 2] ________ [quantidade anual Kg] ________
O que faz com esses resíduos?
□ **não reutilizo**
a. como os descarta? _________
b. em que quantidade anual (kg)? __________
□ **reutilizo**
□ **faço um tratamento para valorizá-los e reutilizá-los para o autoconsumo**
a. que tipo de tratamento faz? _________
b. em que quantidade anual? _________
c. se não for na totalidade, o que faz e como descarta o resto? ______________
□ **faço um tratamento para valorizá-los e vendê-los**
a. que tipo de tratamento faz? _________
b. em que quantidade anual? _________
c. se não for na totalidade, o que faz e como descarta o resto? ______________
□ **Ambas: tratamento para autoconsumo e venda:**
a. que tipo de tratamento faz? _________
b. em que quantidade anual? _________
c. quantidade para autoconsumo _________
d. quantidade para venda _________
e. se não for na totalidade, o que faz e como descarta o resto? ______________

**B)** Consumos anuais do processo de transformação PRODUTO 2:
a. Energia Eléctrica [quantidade anual em KW] ______
b. Energia Térmica via Caldeira
□ lenha [quantidade anual em Kg] ______
□ biomassa [quantidade anual em Kg] ______
c. Água [quantidade anual m³/litros] ______

**C)** Descreva as fases do processo de transformação PRODUTO 2:
Fase 1 [nome] ________________
Fase 2 [nome] ________________
Fase 3 [nome] ________________
Fase 4 [nome] ________________
Fase 5 [nome] ________________
Fase 6 [nome] ________________
Fase 7 [nome] ________________
Fase 8 [nome] ________________

**D)** Descreva o processo de transformação PRODUTO 2:

___________________________________________________________________________________________________________________________________________________________________________________________________________________________________________________________________________________________________________________________________________________________

**Tipo de produto transformado 3 [nome]:** ________________

**PRODUTO TRANSFORMADO 3:**

**A)**
Quantidade anual em Kg? ______________
Tem resíduos e subprodutos da transformação?
□ Sim
□ Não
Se sim, quais são? [nome 3] ________ [quantidade anual Kg] ________
O que faz com esses resíduos?
□ **não reutilizo**
a. como os descarta? _________
b. em que quantidade anual (kg)? __________
□ **reutilizo**
□ **faço um tratamento para valorizá-los e reutilizá-los para o autoconsumo**
a. que tipo de tratamento faz? _________
b. em que quantidade anual? _________
c. se não for na totalidade, o que faz e como descarta o resto? ______________
□ **faço um tratamento para valorizá-los e vendê-los**
a. que tipo de tratamento faz? _________
b. em que quantidade anual? _________
c. se não for na totalidade, o que faz e como descarta o resto? ______________
□ **Ambas: tratamento para autoconsumo e venda:**
a. que tipo de tratamento faz? _________
b. em que quantidade anual? _________
c. quantidade para autoconsumo _________
d. quantidade para venda _________
e. se não for na totalidade, o que faz e como descarta o resto? ______________

**B)** Consumos anuais do processo de transformação PRODUTO 3:
a. Energia Elétrica [quantidade anual em KW] ______
b. Energia Térmica via Caldeira
□ lenha [quantidade anual em Kg] ______
□ biomassa [quantidade anual em Kg] ______
c. Água [quantidade anual m³/litros] ______

**C)** Descreva as fases do processo de transformação PRODUTO 3:
Fase 1 [nome] ________________
Fase 2 [nome] ________________
Fase 3 [nome] ________________
Fase 4 [nome] ________________
Fase 5 [nome] ________________
Fase 6 [nome] ________________
Fase 7 [nome] ________________
Fase 8 [nome] ________________

**D)** Descreva o processo de transformação PRODUTO 3:

___________________________________________________________________________________________________________________________________________________________________________________________________________________________________________________________________________________________________________________________________________________________

**Tipo de produto transformado 4 [nome]:** ________________

**PRODUTO TRANSFORMADO 4:**

**A)**
Quantidade anual em Kg? ______________
Tem resíduos e subprodutos da transformação?
□ Sim
□ Não
Se sim, quais são? [nome 4] ________ [quantidade anual Kg] ________
O que faz com esses resíduos?
□ **não reutilizo**
a. como os descarta? _________
b. em que quantidade anual (kg)? __________
□ **reutilizo**
□ **faço um tratamento para valorizá-los e reutilizá-los para o autoconsumo**
a. que tipo de tratamento faz? _________
b. em que quantidade anual? _________
c. se não for na totalidade, o que faz e como descarta o resto? ______________
□ **faço um tratamento para valorizá-los e vendê-los**
a. que tipo de tratamento faz? _________
b. em que quantidade anual? _________
c. se não for na totalidade, o que faz e como descarta o resto? ______________
□ **Ambas: tratamento para autoconsumo e venda:**
a. que tipo de tratamento faz? _________
b. em que quantidade anual? _________
c. quantidade para autoconsumo _________
d. quantidade para venda _________
e. se não for na totalidade, o que faz e como descarta o resto? ______________

**B)** Consumos anuais do processo de transformação PRODUTO 4:
a. Energia Eléctrica [quantidade anual em KW] ______
b. Energia Térmica via Caldeira
□ lenha [quantidade anual em Kg] ______
□ biomassa [quantidade anual em Kg] ______
c. Água [quantidade anual m³/litros] ______

**C)** Descreva as fases do processo de transformação PRODUTO 4:
Fase 1 [nome] ________________
Fase 2 [nome] ________________
Fase 3 [nome] ________________
Fase 4 [nome] ________________
Fase 5 [nome] ________________
Fase 6 [nome] ________________
Fase 7 [nome] ________________
Fase 8 [nome] ________________

**D)** Descreva o processo de transformação PRODUTO 4:

___________________________________________________________________________________________________________________________________________________________________________________________________________________________________________________________________________________________________________________________________________________________

**Tipo de produto transformado 5 [nome]:** ________________

**PRODUTO TRANSFORMADO 5:**

**A)**
Quantidade anual em Kg? ______________
Tem resíduos e subprodutos da transformação?
□ Sim
□ Não
Se sim, quais são? [nome 5] ________ [quantidade anual Kg] ________
O que faz com esses resíduos?
□ **não reutilizo**
a. como os descarta? _________
b. em que quantidade anual (kg)? __________
□ **reutilizo**
□ **faço um tratamento para valorizá-los e reutilizá-los para o autoconsumo**
a. que tipo de tratamento faz? _________
b. em que quantidade anual? _________
c. se não for na totalidade, o que faz e como descarta o resto? ______________
□ **faço um tratamento para valorizá-los e vendê-los**
a. que tipo de tratamento faz? _________
b. em que quantidade anual? _________
c. se não for na totalidade, o que faz e como descarta o resto? ______________
□ **Ambas: tratamento para autoconsumo e venda:**
a. que tipo de tratamento faz? _________
b. em que quantidade anual? _________
c. quantidade para autoconsumo _________
d. quantidade para venda _________
e. se não for na totalidade, o que faz e como descarta o resto? ______________

**B)** Consumos anuais do processo de transformação PRODUTO 5:
a. Energia Eléctrica [quantidade anual em KW] ______
b. Energia Térmica via Caldeira
□ lenha [quantidade anual em Kg] ______
□ biomassa [quantidade anual em Kg] ______
c. Água [quantidade anual m³/litros] ______

**C)** Descreva as fases do processo de transformação PRODUTO 5:
Fase 1 [nome] ________________
Fase 2 [nome] ________________
Fase 3 [nome] ________________
Fase 4 [nome] ________________
Fase 5 [nome] ________________
Fase 6 [nome] ________________
Fase 7 [nome] ________________
Fase 8 [nome] ________________

**D)** Descreva o processo de transformação PRODUTO 5:

___________________________________________________________________________________________________________________________________________________________________________________________________________________________________________________________________________________________________________________________________________________________

**Tipo de produto transformado 6 [nome]:** ________________

**PRODUTO TRANSFORMADO 6:**

**A)**
Quantidade anual em Kg? ______________
Tem resíduos e subprodutos da transformação?
□ Sim
□ Não
Se sim, quais são? [nome 6] ________ [quantidade anual Kg] ________
O que faz com esses resíduos?
□ **não reutilizo**
a. como os descarta? _________
b. em que quantidade anual (kg)? __________
□ **reutilizo**
□ **faço um tratamento para valorizá-los e reutilizá-los para o autoconsumo**
a. que tipo de tratamento faz? _________
b. em que quantidade anual? _________
c. se não for na totalidade, o que faz e como descarta o resto? ______________
□ **faço um tratamento para valorizá-los e vendê-los**
a. que tipo de tratamento faz? _________
b. em que quantidade anual? _________
c. se não for na totalidade, o que faz e como descarta o resto? ______________
□ **Ambas: tratamento para autoconsumo e venda:**
a. que tipo de tratamento faz? _________
b. em que quantidade anual? _________
c. quantidade para autoconsumo _________
d. quantidade para venda _________
e. se não for na totalidade, o que faz e como descarta o resto? ______________

**B)** Consumos anuais do processo de transformação PRODUTO 6:
a. Energia Eléctrica [quantidade anual em KW] ______
b. Energia Térmica via Caldeira
□ lenha [quantidade anual em Kg] ______
□ biomassa [quantidade anual em Kg] ______
c. Água [quantidade anual m³/litros] ______

**C)** Descreva as fases do processo de transformação PRODUTO 6:
Fase 1 [nome] ________________
Fase 2 [nome] ________________
Fase 3 [nome] ________________
Fase 4 [nome] ________________
Fase 5 [nome] ________________
Fase 6 [nome] ________________
Fase 7 [nome] ________________
Fase 8 [nome] ________________

**D)** Descreva o processo de transformação PRODUTO 6:

___________________________________________________________________________________________________________________________________________________________________________________________________________________________________________________________________________________________________________________________________________________________

***Supplementary figures from KoboToolbox platform***

Figure S1: Distribution of farmer questionnaire responses across study areas. Data from the KoboToolbox platform.

Figure S2. Categories of farmers interviewed. Data from the KoboToolbox platform.

Figure S3. Irrigation adoption among surveyed farms. Data from the KoboToolbox platform.

Figure S4. Type of irrigation adopted by surveyed farmers. Data from the KoboToolbox platform.

Figure S5. Cultivation techniques and level of agricultural mechanisation among interviewed farmers. Data from the KoboToolbox platform.

Figure S6. Post-harvest product management adopted by interviewed farmers. Data from the KoboToolbox platform.

Figure S7. Distribution of agro-processing industries within Nacala and Meconta selected neighbourhoods. Data from the KoboToolbox platform.

Figure S8. Energy use within interviewed agro-processing industries. Data from the KoboToolbox platform.
